# Precise tuning of bacterial translation initiation by non-equilibrium 5′-UTR unfolding observed in single mRNAs

**DOI:** 10.1093/nar/gkac635

**Published:** 2022-07-27

**Authors:** Sujay Ray, Shiba S Dandpat, Surajit Chatterjee, Nils G Walter

**Affiliations:** Single-Molecule Analysis Group, Department of Chemistry and Center for RNA Biomedicine, University of Michigan, Ann Arbor, MI 48109, USA; Single-Molecule Analysis Group, Department of Chemistry and Center for RNA Biomedicine, University of Michigan, Ann Arbor, MI 48109, USA; Single-Molecule Analysis Group, Department of Chemistry and Center for RNA Biomedicine, University of Michigan, Ann Arbor, MI 48109, USA; Single-Molecule Analysis Group, Department of Chemistry and Center for RNA Biomedicine, University of Michigan, Ann Arbor, MI 48109, USA

## Abstract

Noncoding, structured 5′-untranslated regions (5′-UTRs) of bacterial messenger RNAs (mRNAs) can control translation efficiency by forming structures that either recruit or repel the ribosome. Here we exploit a 5′-UTR embedded preQ_1_-sensing, pseudoknotted translational riboswitch to probe how binding of a small ligand controls recruitment of the bacterial ribosome to the partially overlapping Shine-Dalgarno (SD) sequence. Combining single-molecule fluorescence microscopy with mutational analyses, we find that the stability of 30S ribosomal subunit binding is inversely correlated with the free energy needed to unfold the 5′-UTR during mRNA accommodation into the mRNA binding cleft. Ligand binding to the riboswitch stabilizes the structure to both antagonize 30S recruitment and accelerate 30S dissociation. Proximity of the 5′-UTR and stability of the SD:anti-SD interaction both play important roles in modulating the initial 30S-mRNA interaction. Finally, depletion of small ribosomal subunit protein S1, known to help resolve structured 5′-UTRs, further increases the energetic penalty for mRNA accommodation. The resulting model of rapid standby site exploration followed by gated non-equilibrium unfolding of the 5′-UTR during accommodation provides a mechanistic understanding of how translation efficiency is governed by riboswitches and other dynamic structure motifs embedded upstream of the translation initiation site of bacterial mRNAs.

## INTRODUCTION

5′-Untranslated regions (5′-UTRs) of messenger RNAs (mRNA) are essential for regulating protein expression in all cells (1–5). Direct interactions between the small, or 30S ribosomal subunit, and the 5′-UTR allow modulation of initiation as the rate-limiting step of bacterial mRNA translation (5–8). The earliest stage of initiation involves two steps as major contributors to overall translation efficiency: reversible binding of the mRNA to a loosely defined, single-stranded ‘standby site’, followed by short 30S scanning, mRNA unfolding and accommodation into the mRNA binding cleft of the 30S subunit (9–12). The cleft-accommodated 30S ribosomal binding site (RBS) of the mRNA stretches from nucleotides (nt) −18 to + 10 relative to the start site (position +1) (13) and often encompasses a purine-rich region around positions −7 to −4 known as the Shine Dalgarno (SD) sequence (14). This sequence directly engages with the 3′-terminus of the 16S ribosomal RNA (rRNA) of the 30S subunit—the so-called anti-SD sequence—through base pairing. Translation initiation thus requires unfolding of intrinsic mRNA structure near the start codon, imposing a structure-dependent energetic penalty on translation efficiency (14–18). Consequently, transcriptome-wide studies have shown that an increased translation efficiency of a given mRNA generally correlates with its reduced propensity to form secondary structures near the RBS (16,17,19).

While the absence of local secondary structure permits efficient translation initiation on mRNAs that lack an SD sequence (20), structured RNA motifs such as riboswitches embedded in the 5′-UTR require an SD sequence for efficient gene expression, enabling dynamic regulation (15–18,21–23). Primarily found in bacteria, riboswitches are cis-regulatory elements typically controlling either transcription or translation (16–18). Following ligand binding to its aptamer domain, a typical translational riboswitch changes the secondary structure of the downstream expression platform to sequester (part of) the SD sequence, thus repressing translation initiation (19).

**Figure 1.**
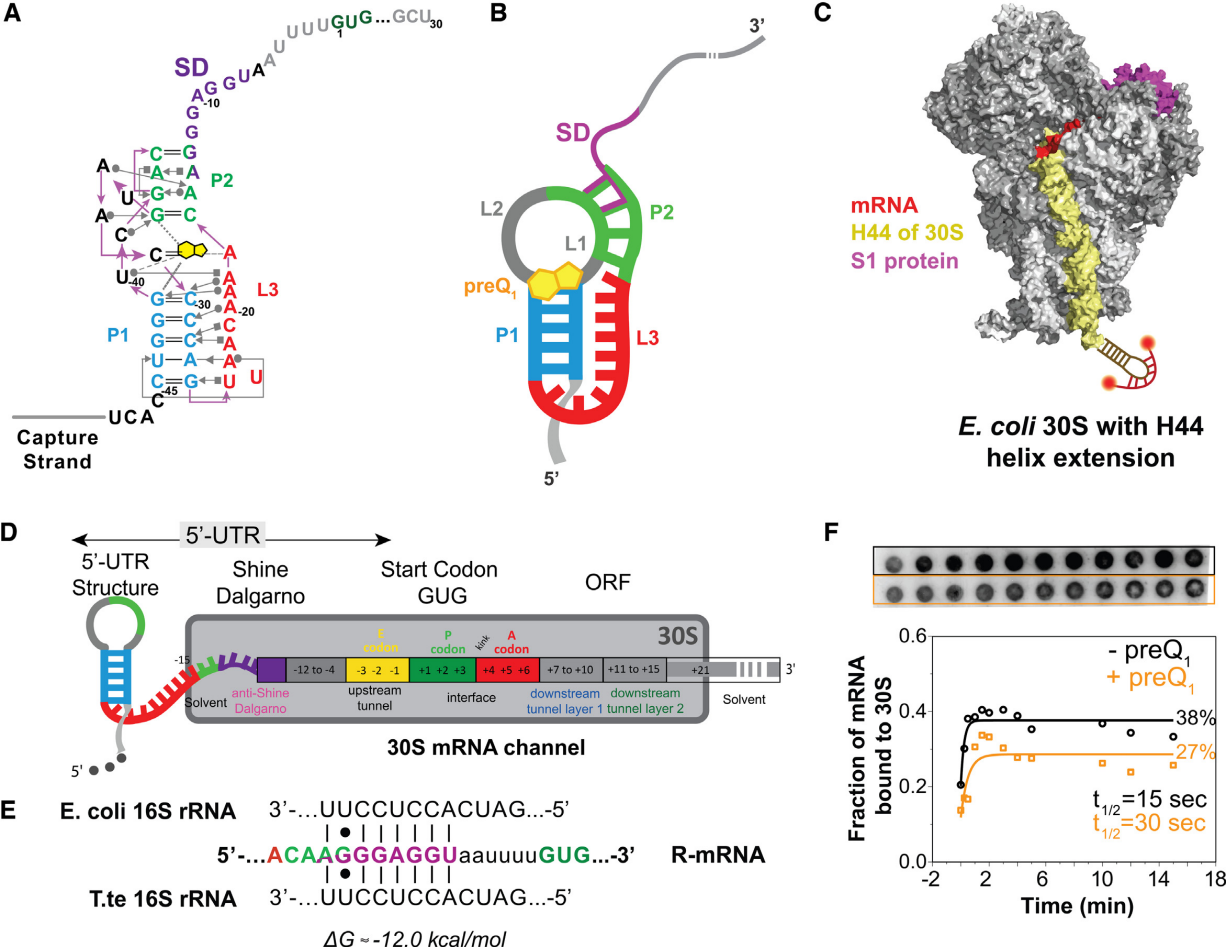
Schematic of a model mRNA with *Tte*-riboswitch at its 5′-UTR to study translation initiation. (**A**) Structural map of the preQ_1_ translational riboswitch from *Tte* displayed with Leontis–Westhof notations (76). The SD sequence (purple) partially overlaps the P2 stem nucleotides (green). (**B**) Schematic diagram of the preQ_1_ bound riboswitch containing mRNA (termed R-mRNA) from *T. tengcongensis* (*Tte*). (**C)** Schematic representation of the mutated ribosome used here, containing a hairpin extension at the helix-44. The extension is hybridized with a dual-labeled complementary DNA oligonucleotide (red). Based on the structural information in Loveland *et al.* (77). (**D**) Schematic diagram of *Tte* R-mRNA and its expected occupancy in the mRNA channel of the 30S subunit during initiation, where +1 is the first nucleotide of the open reading frame. The mRNA channel representation is based on the structural information from (13). (**E**) Base-pairing interactions between the Shine-Dalgarno sequence of our *Tte* mRNA transcript and the 16S rRNA of *E. coli* or *Tte*. (**F**) Autoradiograph of a filter binding membrane measuring the efficiency of 30S–mRNA complex formation as a function of time at zero and 10 μM preQ_1_ concentrations (top). The resulting fraction of bound mRNA is plotted as a function of time (bottom).

The extent of riboswitch-mediated translational control varies across the many riboswitch classes discovered to date. In general, larger and more complex riboswitches may entirely abrogate SD and/or start codon access, leading to switch-like ON/OFF behavior (24,25). In contrast, smaller riboswitches, as exemplified by the H-type pseudoknot of the class-I preQ_1_ (or 7-aminomethyl-7-deazaguanine) riboswitch from the thermophilic bacterium *Thermoanaerobacter tengcongensis* (*Tte*), affect gene expression through only partial SD sequestration (Figure 1) (26–30). Previous *in vitro* isothermal titration measurements (26–30) have observed the temperature dependence of the *Tte* riboswitch, suggesting that it binds ligand with reduced, but still high affinity at its optimal growth temperature (50–80°C). How binding of a small ligand, which yields only little thermodynamic free energy (∼10 kcal/mol) (30,31), regulates mRNA accessibility by the much larger bacterial 30S subunit has remained a conundrum. Transcriptome-wide in vivo SHAPE-MaP analysis of general translation initiation supports a general model wherein an mRNA first binds to a standby site on the 30S subunit (7), followed by transient unfolding of 5′-UTR structure during accommodation into the mRNA cleft, imposing a non-equilibrium energetic penalty on translation initiation (10,11). In the context of riboswitches, these findings suggest that the relative non-equilibrium aptamer unfolding versus 30S binding kinetics may play a gateway role in regulation (32,33), however, the mechanism of this ligand-controlled process has not been observed directly.

Recently, we have demonstrated that 30S binding to a preQ_1_ riboswitch-containing nascent mRNA in a paused transcription elongation complex depends on the mRNA structure, as well as transcription factors (34). Here we focus on the specific role of 5′ mRNA structure on initial 30S recruitment during translation initiation after the transcription complex has passed. To this end, we implement a Single-Molecule Kinetic Analysis of Ribosome Binding (SiM-KARB) assay based on a labeled bacterial 30S subunit to probe 5′-UTR accessibility directly as the control mechanism of translation initiation by the *Tte* preQ_1_ riboswitch. Repetitive binding and dissociation of the 30S subunit to the riboswitch-hosting mRNA (henceforth referred to as ‘R-mRNA’) reveals short binding events that mutational analyses enable us to assign as non-specific ‘standby site’ interactions, whereas significantly longer binding events represent ‘cleft-accommodated’ interactions where R-mRNA becomes partially unfolded and accommodated into the mRNA binding cleft of the 30S subunit. We find that the number of standby and cleft-accommodated binding events are similarly reduced in the presence of preQ_1_ and accordingly their average kinetic parameters change significantly. Strategic mutations in or near the RBS unveil that increasing aptamer-SD distance and SD:anti-SD base pairing strength both favor transient standby site and stable cleft-accommodated binding by the 30S subunit. Finally, the effect of RNA secondary structure near the RBS is further verified by depleting ribosomal protein S1, upon which the 30S subunit shows impaired unfolding of the riboswitch as evidenced by an increased energetic penalty of mRNA accommodation. Taken together, our data support a model wherein the stability of 30S binding is inversely related to the non-equilibrium free energy of aptamer unfolding during mRNA accommodation, enabling the riboswitch ligand to reduce both 30S binding speed and kinetic stability as two distinguishable mechanisms for modulating bacterial translation efficiency.

## MATERIALS AND METHODS

### Initiation complex formation assay

30S ribosomal subunits were prepared and labelled using a previously described protocol (34). 30S initiation complexes were prepared by mixing 1 μM R-mRNA*^FL^* or truncations (R-mRNA*^+30^* and R-mRNA*^–^^11^*), 2 mM GTP, 3 μM each of IF1, IF2 and IF3, 3 μM ^32^P-fMet-tRNA^fMet^, 1 mM MgCl_2_, 1.5 μM twice salt-washed 30S ribosomes, and Tris-polymix buffer, composed of 50 mM Tris-OAc (pH 7.5 at 25°C), 100 mM KCl, 5 mM NH_4_OAc, 0.5 mM Ca (OAc)_2_, 5 mM Mg (OAc)_2_, 6 mM β-mercaptoethanol, 0.5 mM EDTA, 5 mM putrescine and 1 mM spermidine (the total Mg^2+^ concentration in the reaction from all of the added components was ∼7.5 mM). Reactions were incubated in a 37°C water bath for 50 min and then the radioactive counts in 1 μl of the reaction were measured by scintillation counting. Successfully formed 30S ICs were purified away from unincorporated initiator tRNA and initiation factors by carefully layering the sample onto a 1.3 ml sucrose cushion (1.1 M sucrose, Tris-polymix buffer, 15 mM MgCl_2_, 0.5 mM EDTA) in an ultracentrifuge tube, and centrifuged in a Beckman TLA-100.3 rotor at 69 000 rpm for 2.5 h at 4°C. The supernatant was carefully removed, and the pelleted material was resuspended by gentle pipetting in 40 μl of Tris-polymix buffer. The radioactive counts in 1 μl of the resuspended material were measured by scintillation counting and the efficiency of 30S IC formation was calculated by taking the ratio of counts after and before centrifugation.

### Cloning of sequences encoding the R-mRNA and different mutants

The complete mRNA transcript, including the TTE_RS07450 and TTE_RS07445 (TTE1564 and TTE1563, respectively) ORFs and its 30 UTR as predicted from the FindTerm algorithm (Soft-Berry), was amplified using PCR from *T. tengcongensis* genomic DNA, which was purchased from the NITE Biological Resource Center. The amplified region was cloned into the pUC19 plasmid between the BamHI and HindIII sites with an engineered upstream T7 promoter (pUC19_*Tte*). Different lengths of DNA were prepared by PCR amplification of the desired parts of the DNA. DpnI enzyme, which cleaves methylated DNA, is used to digest parent plasmid. 2′-*O*-methylation (2′-OMe) modification in the first two bases of the reverse primers ensures that during transcription in the next step, RNA polymerase dissociate without adding additional bases at the 3′ end of the RNA. Transcription reactions were performed in the presence of 120mM HEPES-KOH (pH 7.5 at 25°C), 30 mM MgCl_2_, 2mM spermidine, 40mM dithiothreitol (DTT), 30mM NTPs, 0.01% (w/v) Triton X-100, 400 nM PCR amplified DNA, 0.01U/ml pyrophosphatase and 0.2 mg/ml T7 RNA polymerase in a total volume of 150 μl. Transcription reactions were incubated at 37°C for ∼18 h. mRNA was purified by denaturing, 7 M urea, PAGE, detected using brief 254-nm ultraviolet radiation and gel-eluted overnight. mRNAs were ethanol-precipitated and resuspended in water.

Different R-mRNA mutations were generated from the original pUC19_Tte plasmid. PCR based site-directed mutagenesis was performed with primers (Supplementary Table S1 for RNA sequences and Supplementary Table S2 for primer sequences) designed to span the mutated bases. RNAs were generated from the plasmids in the same way as described above.

### 3′ Fluorophore labeling of RNA

RNA constructs prepared by transcription as described above were labeled with a Cy3 fluorophore at their 3′ end following a method described previously by Willkomm and Hartmann (35) with several modifications. Briefly, RNA constructs were first oxidized by incubating 5 μM RNA in 100 mM NaOAc (pH 5.2) with freshly prepared 2.5 mM sodium (meta) periodate (Fluka, 71859) on ice for 70 min, protected from light. Subsequently, the oxidized RNA was precipitated with the addition of 0.1 V of 3 M NaOAc (pH 5.2) and 2.5 V of cold absolute ethanol, followed by incubated on dry ice until frozen. The solution was inverted until just thawed and then centrifuged at 20,800 × g for 45 min at 4°C to pellet the RNA. The supernatant was removed by pipetting, and the pellets were then washed with ∼0.3 V of cold 70% (v/v) ethanol and centrifuged again for 20 min. The wash was removed by pipetting and the pellets were dried under vacuum. The oxidized RNA was then coupled with a hydrazide derivative of the fluorophore Cy3 (GE Healthcare, PA13120). A typical 100 μl coupling reaction contained ∼0.2–1.0 nmol of RNA, 50 nmol of Cy3 hydrazide (dye) dissolved in 10 μl of DMSO, and 100 mM NaOAc (pH 5.2). Solutions were degassed prior to the addition of dye, and the headspace above fully assembled reactions was flushed with nitrogen before capping the reaction tube. Reactions were protected from light and incubated at room temperature for 4 hr with agitation. In all subsequent steps, solutions were protected from light. After the end of the incubation, the Cy3-labeled RNA was precipitated with the addition of 0.1 V of 3 M NaOAc (pH 5.2) and 2.5 V of cold absolute ethanol, followed by incubated on dry ice until frozen. The solution was inverted until just thawed and then centrifuged at 20 800 × g for 45 min at 4°C to pellet the RNA. The supernatant was removed by pipetting, and the pellets were then washed with 2 V of cold 70% ethanol and centrifuged again for 20 min. The wash was removed by pipetting and the pellets washed again with 0.5 V of cold 70% ethanol and centrifuged again for 15 min. This final wash was removed by pipetting and the RNA pellets were dried under vacuum and resuspended in 30 μl cold milliQ water, and then desalted using Illustra MicroSpin G-50 columns that had been preequilibrated in milliQ water. The final concentration of RNA in the recovered solution was determined spectrophotometrically using a Nanodrop2000 spectrophotometer, using the respective extinction coefficient at 260 nm (ϵ_260_) for the RNA (945 180 M^–1^ cm^–1^) and ϵ_550_ = 150 000 M^–1^ cm^–1^ for Cy3. The contribution of dye to the absorbance at 260 nm was accounted for as follows: *A*_260_, RNA = *A*_260_ − 0.08 × *A*_550_.

### RNA filter binding assay

The principle for this assay is based on the double-filter method described before, in which the binding of radiolabeled nucleic acids by proteins or other macromolecules is assessed by filter binding reactions through a pair of stacked membranes and measuring the amount of radioactivity retained in each (36–38).

Radiolabeled R-mRNA*^+30^* was prepared in two steps. In the first step, 5 μM RNA was dephosphorylated using Antarctic phosphatase enzyme (New England Biolab). The reaction was incubated at 37°C for 30 min, followed by heat deactivation for 2 min at 80°C. In the second step, dephosphorylated RNA was phosphorylated using ^32^P-labeled ɣ-ATP and T4 Poly Nucleotide Kinase (NEB). The reaction was incubated at 37°C for 30 min. The resulting radiolabeled RNA was purified by a spin column.

Twice salt-washed 30S subunits used for all assays were activated by incubation at 37°C in the Tris-polymix buffer for 5 min immediately prior to use. For each reaction, 3 μl of 3 mM R-mRNA^+30^ was re-folded in the absence and presence of 1 μM preQ_1_ by heating to 70°C for 2 min, followed by slow cooling to room temperature for 15 min. 30S subunits in Tris-polymix buffer was added to the RNA with and without preQ_1_ at different time interval into the filtration column. Membranes were pre-wet in binding buffer for 30 min. A membrane stack was constructed by stacking (from top to bottom): a reinforced nitrocellulose membrane (Optitran BA-S 85, Whatman #10-439-191), a Whatman 1MM filter paper, a positively-charged nylon membrane (BrightStar-Plus, Ambion), and a second Whatman 1MM filter paper. The membrane stack was clamped inside of 96-well dot-blot manifold (Mini-fold, Schleicher & Schuell) and were then washed with cold binding buffer (100 μl per well) and dried by applying vacuum.

The 30S and RNA binding reactions were pipetted into the wells of the manifold, drawn through the membranes under vacuum, and then washed 100 μl cold buffer. Vacuum was applied for 2–3 min until the membranes appeared dry and then membranes were wrapped in saran wrap and imaged using a storage phosphor screen and Typhoon 9410 Variable Mode Imager (GE Healthcare Life Sciences) and quantified using ImageQuant v 5.2 (Molecular Dynamics). Radiolabeled RNA that is successfully incorporated into the 30S–RNA complex is preferentially retained in the nitrocellulose filter, while unbound tRNA is trapped in the positively charged nylon filter. The fraction of bound and unbound 30S were calculated as described previously (37).

### 30S ribosomal subunit purification, labeling and S1 depletion/reconstitution

Mutant *E. coli* pKK3535 plasmid strains with an extension at the helix-44 of the 16S rRNA was generously provided by Joseph Puglisi (Stanford University). This extension allows labeling of the 30S using a DNA oligonucleotide complementary to the extended portion of the helix-44. Salt-washed 30S subunits were prepared using previously described protocols (34,39). 30S labeling was performed with a 10-fold excess of dual-Cy5 labeled DNA oligonucleotide (Supplementary Table S1) as described (34). The final concentration of the 30S subunit in the recovered solution was determined spectrophotometrically using the extinction coefficient ϵ_260_ = 14 492 753.62 M^–1^ cm^–1^.

30S subunits depleted of S1 (ΔS1–30S) were prepared following a method adapted from Lauber *et al* (40). Briefly, because S1 has a high affinity for polyU RNA, it can be efficiently removed from the 30S subunit by incubation with polyU resin. 125 mg of polyuridylic acid–agarose resin (Sigma, P8563) was swelled in 15 ml of polyU wash buffer (20 mM Tris–HCl, pH 7.5 at 25°C, 100 mM NaCl) in a Poly-Prep chromatography column (Bio-Rad, 7311550). All subsequent steps were performed at 4°C on ice to prevent degradation of the polyuridylic acid. The column was placed inside of a 15 ml Falcon tube and centrifuged in a swinging bucket rotor at 100 × g for 1 min. The column was then washed by adding 1 ml of polyU wash buffer and centrifuging for an additional minute. This wash step was repeated 12 times in total to extensively remove loosely bound or degraded polyU RNA from the column. The column was then equilibrated with six 1-ml washes with S1-depletion buffer (20 mM Tris–HCl, pH 7.5 at 25°C, 1 M NH_4_Cl, 10 mM MgCl_2_, 60 mM KCl and 1 mM DTT). Reconstitution was performed with S1 protein purified as described (34,41).

### Single-molecule fluorescence assay

A prism-type total internal reflection fluorescence setup built around an Olympus-IX83 microscope, equipped with 60× 1.20 N.A. water objective and four sCMOS cameras (Hamamatsu, Flash-4 V3) and four different wavelength laser lines was used to perform the ribosome binding experiments (Only two laser lines and two cameras were used). Flow cell sample channels were prepared on surface passivating quartz microscope slides though coated with a mixture of 90% methoxy Poly-Ethelyn Glycol succinimidyl valeric acid (m-PEG SVA) and 10% biotin-PEG succinimidyl valeric acid (biotin-PEG SVA) using previously established protocols (42,43). For surface immobilization of R-mRNA, the sample chamber was treated with 0.2 mg/ml streptavidin to bind to the biotin from the PEG. Solution containing ∼50 pM previously annealed biotinylated capture strand-RNA complex was introduced to the chamber to sparsely coat the PEG surface with streptavidin. Excess non-immobilized RNAs were then washed with 200 μl wash buffer (10 mM Tris Base, pH 7.5 @ 25°C). Steady-state SiM-KARB and ribosome binding measurements were performed by first forming the riboswitches by incubating the surface-immobilized RNA constructs with at a given concentration of preQ_1_ in Tris-polymix buffer for 15 min. 20 nM concentration of dual Cy-5 labeled ribosome solution with same concentration of preQ_1_ was then added to the chamber in an imaging solution (Tris-polymix buffer, 5 mM protocatechuic acid and 50 nM protocatechuate-3,4-dioxygenase, 2 mM Trolox). The dual labeling ensured long binding events of 30S to R-mRNA despite photobleaching of individual Cy5 molecule. Typically, three independent biochemical replicates were performed, and all data were pooled together for further analysis. For control experiments to test the 30S binding specificity, biotinylated capture strand DNA labeled with 5′-Cy3 was immobilized to the slide surface without any R-mRNA. An integration time of 100 ms was used unless otherwise specified for the experiments. A combination of continuous and shuttered illumination was used to capture slow and fast dynamic events, respectively. Shuttered illumination was specifically used for non-equilibrium ligand-jump experiments. Ten thousand frame movies at the rate of 100 ms per frame (150 ms per frame for mutants due to long observed binding times of 30S to mutants) were recorded for each condition with continuous 532 and 639 nm laser sources for the whole duration.

### Ligand-jump experiment

Surface immobilized R-mRNA*^+30^* molecules in the absence of preQ_1_ were first introduced to buffer containing 20 mM of Cy5 labeled 30S and monitored for eight hundred seconds, followed by 600 s dark time (shown in gray, Figure 3A). During the dark period, a fresh buffer solution containing 1 μM preQ_1_ and same concentration of Cy5-30S as before was injected. The dark period allowed time for the homogeneous exchange of buffer and OSS to reduce photobleaching probability. After the dark period, molecules in the same field of view were tracked in real-time for another eight hundred seconds in the presence of the preQ_1_ without altering the preexisting concentration of buffer and 30S in the solution.

### Analysis of single-molecule data

Single-molecule time traces were generated by a custom-written MATLAB code. Furthermore, custom analysis programs in MATLAB were used to extract statistical data from individual molecules, and finally Origin Pro9 was used to plot the data.

## RESULTS

### Riboswitch ligand modulates 30S binding to the mRNA 5′-UTR

Prior crystallographic and single-molecule FRET studies of its isolated aptamer domain have suggested that the *Tte* preQ_1_ riboswitch controls translation initiation by the ligand-induced sequestration of two nucleotides shared between the P2 helix of the pseudoknot and the SD sequence (29,30,41,44) (Figure 1A and B). The 30S subunit (Figure 1C), however, occupies a total of ∼30 nt in the 5′-UTR that unfold when stably accommodated into the mRNA binding cleft and are thus considered part of the expression platform (Figure 1D). We transcribed a *Tte* gene-1564 sequence that includes this entire expression platform under the influence of the preQ_1_ riboswitch, either as a full-length mRNA (R-mRNA*^FL^*) or as shorter nascent RNA sequences as would emerge during transcription as a platform for binding the first 30S subunit that initiates the pioneering round of translation (Figure 1D) (28). Exploiting the high sequence conservation and comparable thermodynamics of the SD:anti-SD interactions between *Tte* and *Escherichia coli* (*Eco*; Figure 1E), we used purified *Eco* 30S subunits for comparing full initiation complex (IC) formation efficiencies on R-mRNAs of different lengths. As expected, we found similar efficiencies for R-mRNA*^FL^* and the RNA truncated 30 nt downstream of the start codon (R-mRNA*^+30^*), but observed no IC formation on the RNA lacking both SD sequence and start codon (R-mRNA*^–^^11^*; Supplementary Figure S1), supporting the specificity of the 30S subunit interaction. To focus on the role of the 5′-UTR in controlling access of the 30S subunit without the undesired potential for binding further downstream, we therefore chose the short, initiation-competent R-mRNA*^+30^* for our studies.

To characterize the 30S binding efficiency of R-mRNA*^+30^* in response to the addition of preQ_1_ ligand, we performed a radioactive filter-binding assay (38), wherein the fraction of 30S-bound over total ^32^P-radiolabeled R-mRNA*^+30^* is calculated from the blotted, double-filtered dots (Figure 1F). A time course of the normalized 30S-mRNA complex formation in the absence and presence of a high concentration (10 μM) (28) of preQ_1_ revealed an ∼2-fold adverse effect of preQ_1_ on both pre-steady state kinetics and endpoint (Figure 1F). This level of modulation is very similar to earlier findings that the translation of R-mRNA*^FL^* is decreased *in vitro* by ∼40% at saturating preQ_1_ concentration (28), supporting the notion that the ligand significantly affects translation at the initial 30S binding step. In-cell gene reporter assays on two other translational riboswitches, a class-II preQ_1_riboswitch and an adenine riboswitch, both show similarly modest ∼3-fold increases in gene expression (45,46). The molecular mechanism of this effect, however, is not revealed by such ensemble assays.

### Riboswitch ligand both antagonizes 30S recruitment and accelerates 30S dissociation

**Figure 2.**
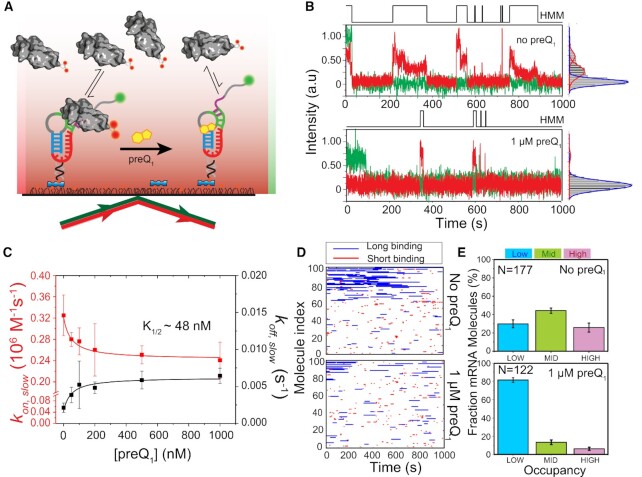
preQ_1_-dependent 30S binding to R-mRNA at the single-molecule level. (**A**) Schematic representation of our SiM-KARB assay. 3′-Cy3 labeled R-mRNA molecules were immobilized on a slide surface. Repeated binding and dissociation of 30S subunit (dual-labeled with Cy5) was monitored through the co-localization of Cy3 and Cy5 fluorescence. (**B**) Representative R-mRNA^+30^ (green) and 30S (red) fluorescence intensity versus time trajectories and corresponding intensity histograms for a single R-mRNA^+30^ molecule in the absence (top panel) or presence (bottom panel) of preQ_1_. HMM idealizations to a two-state model (black, top) were plotted as a function of time. (**C**) 30S-binding (*k*_on,slow_, red) and dissociation (k_off,slow_; black) rate constants were determined from exponential fits of dwell times in the unbound and bound states, respectively, as a function of preQ_1_ concentration. The corresponding half-saturation (*K*_1/2_) values were determined by non-cooperative Hill equation fitting with Hill coefficients of −1 and +1, respectively. Error bars were obtained by bootstrapping the data as explained in Supplementary Information. (**D**) Rastergram of 100 randomly selected traces of 30S binding to individual R-mRNA^+30^ molecules in the absence (top panel) and presence (bottom panel) of preQ_1_. 30S binding events on R-mRNA^+30^ were represented in either red (short or standby events) or blue (long or cleft-accommodated events). (**E**) Histograms of the fractional bound time for which individual R-mRNA^+30^ molecules were occupied by 30S subunits in the absence (top) or presence (bottom) of preQ_1_. All 30S-bound R-mRNA^+30^ molecules were empirically ranked as low (<0.1 fractional bound time, cyan), mid (0.1 to 0.2 fractional bound-time, green), or high (>0.2 fractional bound time, pink) in 30S occupancy. *N* represents the total number of molecules included for each condition.

To directly evaluate 30S binding to R-mRNA*^+30^* as an important gateway to initiating translation, we developed SiM-KARB, wherein a purified *Eco* 30S subunit with an extended 16S rRNA helix-44 is fluorescently tagged (39) by hybridization with a doubly Cy5-labeled (to mitigate photobleaching) DNA oligonucleotide (Figure 1C). Repeated binding and dissociation of an excess of these 30S subunits in solution (red) to surface-immobilized 3′-Cy3 labeled R-mRNA*^+30^* (green) was monitored by total internal reflection fluorescence (TIRF) microscopy as transient diffraction-limited co-localization events, performed in the 50 mM Tris-polymix buffer routinely used in translation assays (34,47) (Figure 2A, and B; see Materials and Methods for details). A mixture of short and long binding events was observed with the presence of preQ_1_ modulating the events (Figure 2B), supporting two main distinguishable classes of 30S-mRNA interactions, consistent with the well-established standby and cleft-accommodated binding modes, respectively. In contrast to the presence of R-mRNA*^+30^*, no characteristic repeated 30S binding events were observed in the absence of mRNA, further ruling out non-specific surface interactions of the labelled 30S subunit and underscoring the specificity of 30S binding to R-mRNA*^+30^* (Supplementary Figure S2).

To obtain more quantitative, comparative insights, we pursued several layers of further analysis. First, individual time traces obtained by folding and equilibrating R-mRNA*^+30^* in the presence of varying concentrations of preQ_1_ were idealized into two-state Hidden Markov Models (HMM, Figure 2B). The HMMs were then used to extract the dwell times during which 30S was either unbound (*τ_unbound_*) or bound (*τ_bound_*), which both were best fit globally with double-exponential functions (48,49) revealing each two rate constants: 30S binding rate constants *k_on,fast_* and *k_on,slow_*, and 30S dissociation rate constants *k_off,fast_* and *k_off,slow_*, respectively. Of these, the slow *k_on,slow_* and *k_off,slow_* represented ∼30–60% and ∼70–90%, respectively, of all binding events and were found to be most significantly affected by the addition of preQ_1_, with *k_on,slow_* decreasing and *k_off,slow_* increasing (Figure 2C, Supplementary Figure S3 and Table S3A, B). Of note, while dual Cy5-labeling prolonged the observation of bound 30S subunits, we additionally corrected all rate constants for residual photobleaching of both fluorophores (Supplementary Figure S4). The observed ∼27% deceleration of 30S binding with increasing preQ_1_ (from *k_on,slow_* = 0.33 ± 0.04 × 10^6^ M^–1^s^–1^ of relative amplitude = 89 ± 4%; to 0.24 ± 0.03 × 10^6^ M^–1^ s^–1^ of 96 ± 8%; Figure 2C, Supplementary Figure S5 and Table S3A) is consistent with a model of partial SD sequestration upon preQ_1_-induced folding of the riboswitch pseudoknot. Complementarily, the ∼3-fold acceleration of 30S dissociation (from *k_off,slow_* = 0.002 ± 0.001 s^–1^ of relative amplitude 58 ± 8%; to 0.006 ± 0.002 s^–1^ of 41 ± 8%; Figure 2C, Supplementary Figure S5, Supplementary Figure S6 and Table S3A) suggests an additional effect of preQ_1_ on dissociation of the assembled 30S−R-mRNA*^+30^* complex. That is, preQ_1_ mediated SD sequestration has two discernable effects on the 30S-mRNA interaction; it suppresses 30S binding and expels 30S once bound. Global fitting of these two ligand dependencies with non-cooperative Hill equations yielded a half-saturation point of ∼48 nM preQ_1_ (Figure 2C), in reasonable agreement with measurements of ligand binding to the isolated riboswitch (29,30) and demonstrating that R-mRNA*^+30^* is saturated at 1 μM preQ_1_.

Second, we asked how the 30S binding kinetics change for individual R-mRNA^+30^ molecules when folded with 1 μM preQ_1_ present. To this end, individual trace HMMs were arranged into rastergrams sorted by their relative accessibility to the 30S subunit over the entire trace (Figure 2D). Based on a threshold bound time derived from the *k_off,fast_* and *k_off,slow_* dissociation rate constants (Materials and Methods), we categorized individual binding events in the rastergram as either short (red) or long (blue) (Figure 2D). These rastergrams indicate that each R-mRNA*^+30^* molecule interacts quite distinctly with 30S subunits and shows long-term retention of its 30S binding behavior. As expected from an SD sequestration model, the number of total 30S binding events decreases upon addition of saturating preQ_1_ ligand, by ∼17% (Figure 2D). Moreover, while the shorter binding events were less affected (∼6%), the long binding events were reduced by ∼33% in the presence of saturating preQ_1_ (Supplementary Table S4), suggesting that the longer, cleft-accommodated binding of the 30S are primarily disfavored by preQ_1_ induced riboswitch folding.

Lastly, inspired by previous studies equally suggesting that 30S occupancy behaviors of individual mRNA molecules may be retained long-term, in aggregate leading to a significant downregulation of protein expression (28), we plotted probability histograms that bin the total fraction of time single R-mRNA*^+30^* molecules remain 30S associated within our experimental time window of 1000 s (Supplementary Figure S7). To classify individual R-mRNA behaviors based on their accessibility, we combined all datasets into a histogram wherein three Gaussian probability distributions were resolved, representing distinct levels of accessibility (Supplementary Figure S7A). A 30S occupancy ranking of individual molecules as low (L, fractional 30S bound time < 0.10), medium (M, fractional 30S bound time between 0.10 and 0.20) or high (H, fractional 30S bound time > 0.20) was derived based on the crossover points of those Gaussians (Supplementary Figure S7A). In the absence of preQ_1_, 30 ± 4%, 44 ± 3% and 26 ± 5% of molecules show L, M and H occupancy, respectively; whereas upon addition of saturating 1 μM preQ_1_, a large majority of the molecular population (81 ± 2%) exhibits L occupancy, at the expense of both the M (13 ± 2%) and H (6 ± 2%) occupancies (Figure 2E), supporting the notion that the transition from standby to cleft-accommodated 30S binding is suppressed by the ligand.

Next, control measurements were performed to establish the level of mRNA occupancy in the absence of the 5′-UTR structure. In this measurement, the capture strand was extended to form an RNA-DNA duplex that blocks P1 helix formation and as a result disrupts pseudoknot formation. As expected, mRNA accessibility was significantly increased when pseudoknot formation is thereby suppressed; 52% of R-mRNA*^+30^* molecules now show high occupancy (H), while 24% each display M and L occupancy (Supplementary Figure S8). This measurement establishes that suppression of the 5′-UTR structure significantly increases the accessibility of R-mRNA*^+30^* for 30S.

These findings suggest that individual molecules belong to subpopulations that respond individually to the presence of preQ_1_, with a common trend by which 30S binding becomes suppressed by preQ_1_, thereby lowering the probability of cleft-accommodation of a standby-site bound mRNA as required for translation initiation. Our data thus support a model involving two binding interactions between R-mRNA*^+30^* and 30S subunits, with weak standby site binding and more stable (longer-lived) binding events upon cleft accommodation, the latter of which is more profoundly impacted by the presence of preQ_1_. Finally, the ligand not only competes with 30S for binding to the RNA pseudoknot, but also actively accelerates dissociation of 30S subunits already bound to R-mRNA*^+30^*.

### The riboswitch-hosting mRNA dynamically adapts to changes in preQ_1_ concentration

**Figure 3.**
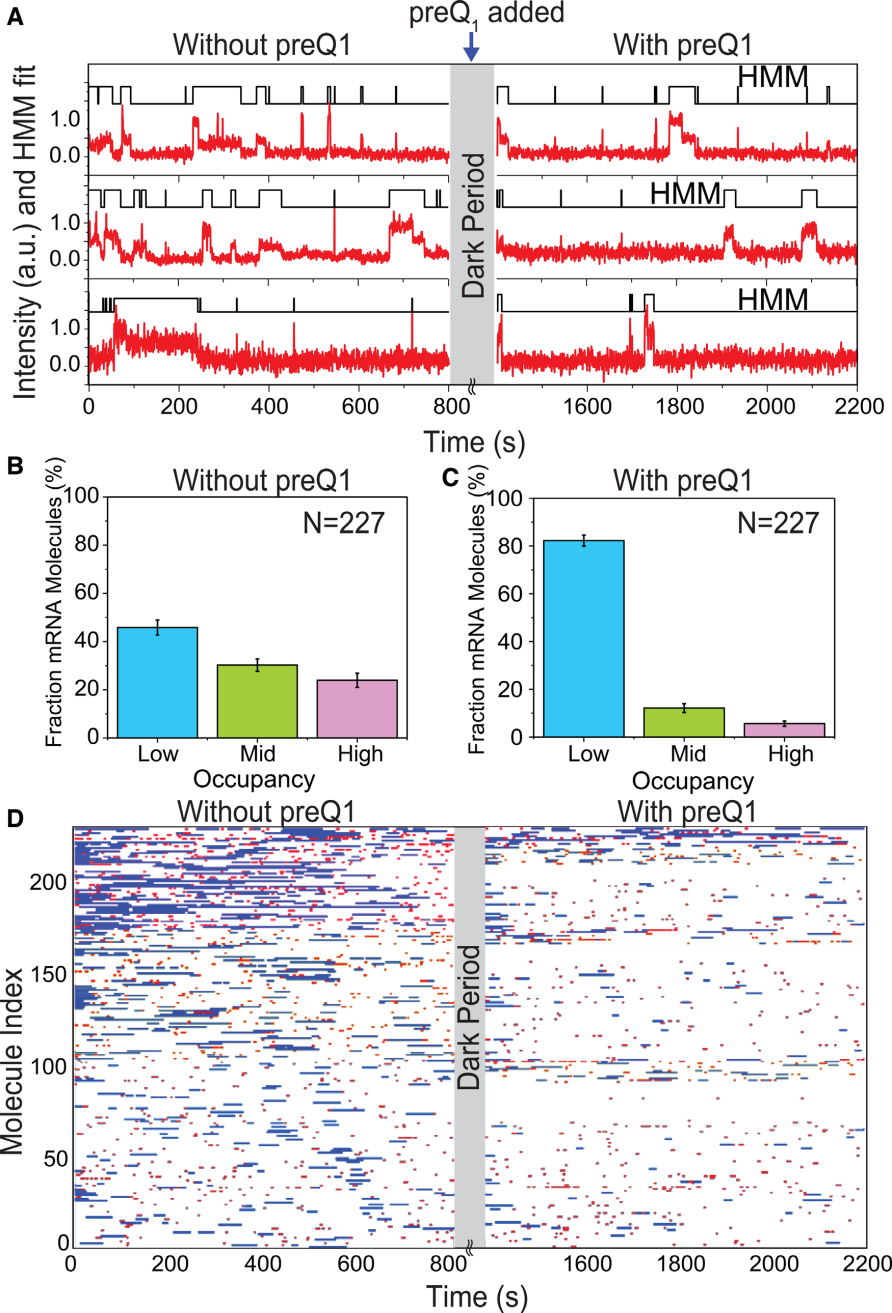
Single mRNA molecules undergo conformational switching upon introduction of preQ_1_ ligand in situ. (**A**) Exemplary single-molecule trajectories from non-equilibrium ligand-jump experiments composed of segments before and after the jump. 30S binding to the same set of individual R-mRNA^+30^ was monitored first in the absence of preQ_1_ (left, −preQ_1_), then in the presence of 1 μM preQ_1_ (right, +preQ_1_). The gray axis break represents a 600 s dark period between segments during which the buffer was exchanged. (**B, C**) Histograms of the fraction of time individual R-mRNA^+30^ molecules are bound by 30S before (B) and after (B) preQ_1_ is introduced. Most molecules shift towards lower occupancy upon introduction of preQ_1_. N, number of mRNA molecules. (**D**) Rastergram displaying the 30S binding behaviors of individual R-mRNA^+30^ molecules before and after introducing preQ_1_. 30S binding events on each R-mRNA^+30^ are represented in red (short or standby events) or blue bar (long or cleft-accommodated events).

The persistence of individual R-mRNA*^+30^* in their equilibrium interaction with 30S subunits raises the question to what extent 30S binding to R-mRNA*^+30^* changes in response to sudden, non-equilibrium variations in its environment, as may occur in the bacterial cells due to, e.g. environmental stresses (50). To address this question, we used a non-equilibrium preQ_1_ ‘ligand-jump’ experiment from zero to saturating ligand while tracking 30S binding (Figure 3A, Materials and methods) (28,51). Consistent with our equilibrium experiments, upon preQ_1_ addition we observed a general reduction in 30S binding frequency (Figure 3A, top and middle) and a decrease in the 30S bound time (Figure 3a, bottom). Overall, the resulting 30% decrease in *k_on__,slow_* and ∼ 3-fold increase in *k_off__,slow_* (Supplementary Figure S9) suggest that preQ_1_ binding *in situ* has similar effects on 30S association with R-mRNA*^+30^* as does equilibrium binding of preQ_1_, both antagonizing 30S recruitment and accelerating dissociation.

Next, we analyzed the probability distribution of 30S fractional bound times. In the absence of preQ_1_, 46 ± 3% of R-mRNA*^+30^* molecules showed L-type 30S occupancy, while 30 ± 3% and 24 ± 3% of molecules showed M and H accessibility, respectively (Figure 3B). By contrast, after addition of saturating preQ_1_ mRNA molecules predominantly exhibited reduced accessibility (82 ± 2% L, 12 ± 2% M and 6 ± 1% L, Figure 3C). This shift from high to low 30S occupancy of the same molecules in response to preQ_1_ further supports that dynamic ligand binding induces the same repulsion and destabilization of 30S subunit binding observed under equilibrium conditions.

Finally, a rastergram allowed us to directly compare the mRNA accessibility in the absence and presence of preQ_1_ (Figure 3D). 48% of molecules responded to the addition of preQ_1_ with a significant reduction in 30S occupancy (Supplementary Table S5A). We also found that the short standby site interactions were reduced by ∼30%, whereas the longer cleft-accommodated interactions were reduced even more profoundly, by ∼60%, underscoring the significant effect of preQ_1_ on particularly cleft-accommodated interactions (Supplementary Table S5B). Still, 46% of molecules remained in the same 30S occupancy rubric and did not significantly respond to the addition of preQ_1_. Only a small (∼6%) population transitioned from a lower to a higher 30S occupancy. The latter observation suggests that, under non-equilibrium conditions, preQ_1_ in some cases may promote the refolding of an mRNA, as was observed previously (28). On the other end of the behavioral spectrum, some 15% of R-mRNA*^+30^* molecules became completely inaccessible to 30S binding.

Overall, these observations underscore the dynamic nature of the significant response of the riboswitch to ligand binding, rendering 30S binding and thus translation efficiency highly adaptive to environmental cues.

### Riboswitch pseudoknot proximity and SD sequence strength independently control 30S binding

In bacteria, the SD sequence is not obligatory for translation initiation (52,53). While only 0.7% of mRNAs are thought to be leaderless in *E. coli* (54), other bacteria harbor up to 72% leaderless mRNAs (55). The preQ_1_ riboswitch and the SD sequence overlap such that preQ_1_ binding promotes formation of the P2 helix of the aptamer, sequestering two nucleotides of the SD sequence (30) (Figure 1A, B). Taken together, these observations raise the question of how sequestering only 2 nt can have the profound effect on 30S binding we observe (30). To address this question, we next created a systematic set of R-mRNA*^+30^* mutants that systematically decouple the aptamer and SD sequence.

**Figure 4.**
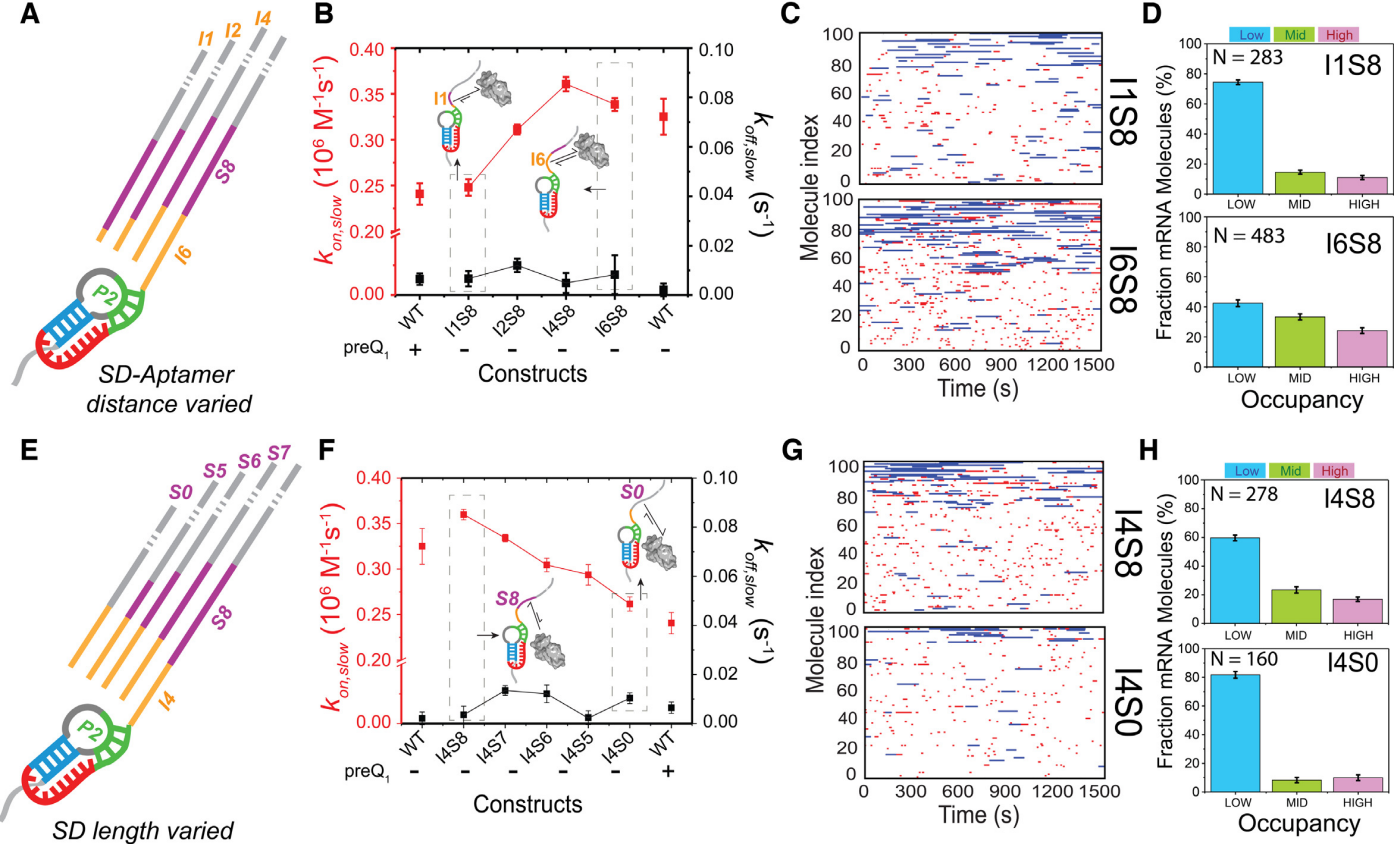
Mutation of the 5′-UTR structure by varying SD-aptamer distance (I) and SD length (S) affects 30S binding. (**A**) Schematic representation for varying the SD-aptamer distance from insert length of one base (I1) to six bases (I6). (**B**) 30S-binding (*k_on,slow_*, red) and dissociation (*k_off,slow_*, black) rate constants for different SD-aptamer distances. Data for WT R-mRNA with and without preQ_1_ are shown for reference. (**C**) Rastergram of 100 randomly selected traces of individual mutant R-mRNA*^+30^* molecules with a 1-nt (I1S8, top panel) or 6-base insert (I6S8, bottom panel). 30S binding is represented in red (short binding events or standby events) or blue (long binding events or cleft-accommodated events). (**D**) Histogram of the fractional bound time for individual I1S8 (top) and I6S8 (bottom) mutant R-mRNA molecules. (**E**) Schematic representation for varying the SD:anti-SD interaction length from full complementarity (S8) to no complementarity (S0) with a fixed number of 4 nt (I4) between the aptamer and SD sequence. (**F**) 30S binding (*k_on,slow_*, red) and dissociation (*k_off,slow_*, black) rate constants for different SD:anti-SD complementarities. (**G**) Rastergram of 100 randomly selected traces of individual mutant R-mRNA*^+30^* molecules with fully available SD (I4S8, top panel) or no SD (I4S0, bottom panel). (**H**) Histogram of the fractional bound time for individual I4S8 (top) and I4S0 (bottom) mutant R-mRNA molecules.

In the first set, the aptamer and SD sequence were increasingly separated by inserting 1–6 nt between them while maintaining the 8-nt long wild-type (WT) SD sequence (Figure 4A, constructs I1S8-I6S8; I1S8, for example, represents a 1-nt insert and an 8-nt long SD sequence; Supplementary Table S1 for the list of sequences). A second set of mutations aimed to increasingly weaken the SD:anti-SD interaction by shortening the SD length from 8 to zero nt while maintaining an unstructured 4-nt UAUA insertion between the aptamer and the remaining SD (Figure 4E,constructs I4S8 to I4S0; I4S0, for example, represents a 4-nt insert and zero SD sequence; Supplementary Table S1 for the list of sequences). All mutants retained all nucleotides participating in the pseudoknotted aptamer structure, and the WT would be annotated I2S8 in this nomenclature.

Strikingly, using our equilibrium SiM-KARB assay, we found a *k_on__,slow_* of 0.25 ± 0.009 × 10^6^ M^–1^s^–1^ for I1S8 in the absence of preQ_1_, within error the same value as the WT displays at saturating preQ_1_ (0.24 ± 0.01 × 10^6^ M^–1^s^–1^; Figure 4B and Supplementary Table S6). This mutant I1S8, as well as I4S8, showed only a minimal effect of preQ_1_ on the 30S binding kinetics (Supplementary Figure S10, Supplementary Table S7 for I1S8 and Supplementary Table S8 for I4S8). That is, when the aptamer is entirely decoupled (removing any overlap) and separated by just 1 nt from the canonical 8-nt SD sequence, it represses 30S binding independently of preQ_1_ as efficiently as the WT only does when fully folded with preQ_1_. Since the aptamer's P2 helix forms only partially in the absence of preQ_1_ (30), this repression does not depend on occlusion of part of the SD sequence, but instead must arise from the steric hindrance the pre-folded, preQ_1_-free (or apo) aptamer structure exerts on the 30S subunit. In support of this hypothesis, *k_on,slow_* values of 0.36 ± 0.006 × 10^6^ M^–1^s^–1^ and 0.34 ± 0.007 × 10^6^ M^–1^s^–1^ were recovered in the absence of preQ_1_ once the SD was moved 4 and 6 nt apart from the SD in mutants I4S8 and I6S8, respectively (Figure 4B, Supplementary Figure S10 and Table S6), equivalent to the value of 0.33 ± 0.02 × 10^6^ M^–1^ s^–1^ for the WT without preQ_1_. A mutant of intermittent 2-nt distance (I2S8) showed an intermediate increase of *k_on,slow_*, also in the absence of preQ_1_ (Figure 4B, Supplementary Figure S10 and Supplementary Table S6), suggesting that the 30S subunit is highly sensitive to the precise distance of the partially formed P2 helix from the SD sequence. A rastergram plot confirmed this trend with an ∼240% increase in short standby binding events and an ∼7% increase in long binding events of I6S8 relative to I1S8, showing that as the SD sequence is moved away from the steric hindrance by the aptamer, it became more accessible for 30S to bind (Figure 4C, Supplementary Table S9 and Table S10). Similarly, the 30S occupancy of single mRNA molecules was low with the aptamer placed proximal to the SD sequence in I1S8 (11 ± 1% H, 15 ± 1% M and 74 ± 1% L occupancy; Figure 4D) and increased when the aptamer was more spaced out, as in I6S8 (24 ± 2% H, 33 ± 2% M and 43 ± 2% L; Figure 4H).

Taken together, these observations reveal that the pre-folded apo-aptamer represses 30S association sterically during the earliest stage of translation initiation, whereas spacing the aptamer's P2 helix as little as 4 nt from the SD sequence alleviates this steric hindrance of 30S binding entirely. This finding reveals a surprisingly limited role of the SD sequence itself in effecting riboswitch-mediated repression of translation initiation. Notably, the dissociation rate constants *k_off,slow_* of this entire set of mutants from I1S8 to I6S8 in the absence of preQ_1_ remains elevated, closer to that of the WT in the presence of preQ_1_ than its absence (Figure 4B), suggesting that the mRNA−30S complex, once formed, may be sensitive to secondary structure of the mRNA even further upstream.

To understand the role of the SD sequence, if any, we investigated our second set of mutants, I4S8 to I4S0 (Figure 4E, Supplementary Figure S11 and Table S6). Using our SiM-KARB assay under equilibrium conditions in the absence of preQ_1_, we found I4S8’s *k_on,slow_* value of 0.36 ± 0.006 × 10^6^ M^–1^s^–1^ to decrease to 0.26 ± 0.008 × 10^6^ M^–1^s^–1^ for I4S0 as the number of complementary SD:anti-SD base pairs decreased from eight to zero (Figure 4F, Supplementary Figure S11 and Table S8). This ∼35% decrease in binding rate constant, and gradual decrease at intermediate SD sequence lengths (Figure 4F), underscores that the mRNA’s SD sequence is an important 30S recruiting factor. Notably, the 30S subunit still binds to I4S0 even in the entire absence of the SD sequence, which corroborates the notion that leaderless mRNAs can function in translation (52,53). We also observed that the *k_on,slow_* for I4S0 in the absence of preQ_1_ comes close to that of the WT in its presence (Figure 4F), providing evidence that preQ_1_ binding to the WT aptamer suppresses access to the SD sequence completely, even though the 30S subunit can still bind elsewhere on our short mRNA. Of note, the dissociation rate constants *k_off,slow_* of this set of mutants from I4S8 to I4S0 in the absence of preQ_1_ again remains closer to the elevated value of the WT in the presence of preQ_1_ (Figure 4F), consistent with the notion of mRNA-30S complex sensitivity to proximal mRNA secondary structure. A comparison of the rastergrams for I4S8 and I4S0 showed that, while the cleft-accommodated (long) binding events were reduced by ∼52%, the short standby bindings were reduced, by ∼45% (Figure 4G, Supplementary Table S11). Finally, the fractional 30S occupancy histograms showed that the mRNA accessibility progressively decreases from 17 ± 2% H, 23 ± 2% M, and 60 ± 2% L population for I4S8 to 10 ± 2% H, 8 ± 2% M, and 82 ± 2% L for I4S0 (Figure 4H).

Taken together, our observations from mutations designed to systematically uncouple aptamer and SD sequence effects indicate that (53) the absence of steric hindrance from proximal secondary structure and a strong SD:anti-SD interaction are two main, independent 30S recruitment factors. That is, while preQ_1_ binding to the aptamer only sequesters two nucleotides of the SD sequence, the riboswitch affects 30S recruitment through both partial occlusion of the SD sequence upon ligand binding and, unexpectedly, its inherent secondary structure independent of ligand.

### Ribosomal protein S1 unfolds the riboswitch to both facilitate and stabilize 30S binding

**Figure 5.**
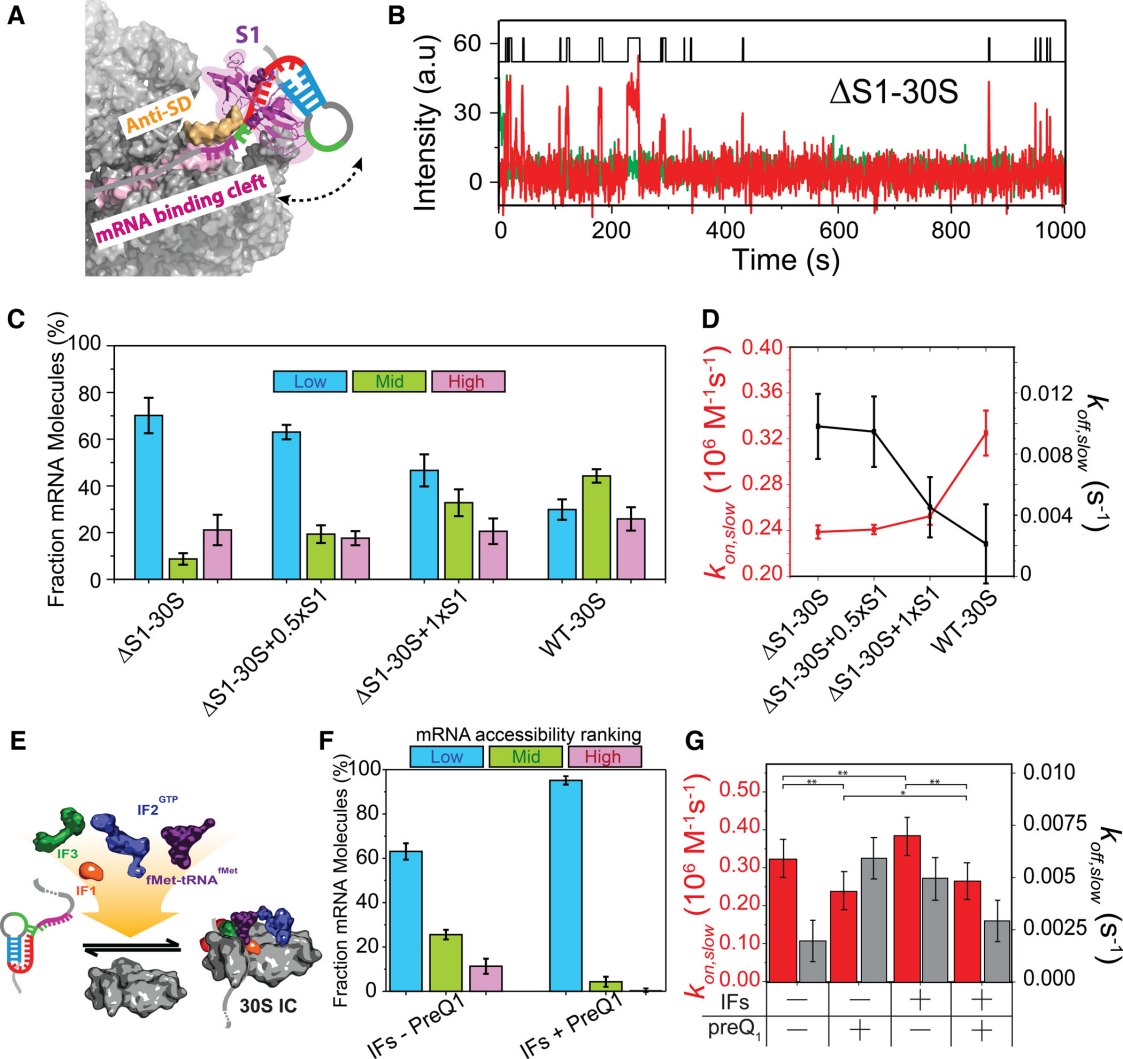
S1 mediates unfolding of the 5′-UTR to enhance and stabilize 30S binding. (**A**) Schematic of S1 mediated unfolding of the 5′-UTR of R-mRNA*^+30^*. Based on the structural information in Loveland *et al.* (77). (**B**) Representative R-mRNA*^+30^* (green) and associated ΔS1–30S (red) binding time trajectory. An HMM idealization to a two-state model (black, top) is plotted as a function of time. (**C**) Histogram of the fractional bound time for individual R-mRNA molecules occupied by for depleted ΔS1–30S (*N* = 71), ΔS1–30S with 50% stoichiometric purified S1 (ΔS1−30S + 0.5×S1) (*N* = 122), ΔS1–30S with fully stoichiometric purified S1 (ΔS1–30S + 1 × S1) (N = 104), and WT-30S. (**D**) Corresponding binding (*k_on,slow,_* red) and dissociation (*k_off,slow,_* black) rate constants. Data for WT R-mRNA without preQ_1_ are shown for comparison. (**E**) Schematic of pre-initiation complex formation in the presence of initiation factors and initiator tRNA. (**F**) Histogram of the fractional bound-time for individual R-mRNA*^+30^* in the presence of all initiation factors and fMet-tRNA^fMet^, in the absence (top) and presence (bottom) of preQ_1_. (**G**) Comparison of binding (*k_on,slow_*) and dissociation (*k_of__f,slow_*) rate constants in the presence or absence of initiation factors, fMet-tRNA^fmet^ and preQ_1_, as indicated. Statistical significance was determined by Student's *t*-test as *P< 10^–2^; **P< 10^–4^.

Our data so far have shown that preQ_1_ binding to WT R-mRNA*^+30^* reduces SD sequence accessibility and thereby both slows and destabilizes 30S binding. Ribosomal protein S1 is known to enhance 30S binding by many mRNAs with structured 5′-UTRs (56,57). The *E. coli* ribosomal protein S1 has six domains. While the two N-terminal domains (D1 and D2) are mainly responsible for binding to the 30S subunit, the four C-terminal domains (D3−D6) bind the mRNA. Recent NMR studies reported that OB-fold domains D3 and D4 provide the main mRNA binding platform, whereas the RNA chaperone activity is related to the conformational dynamics of domain D5 (58). By comparison, protein S1 from *Tte* (*Tte*-S1) is significantly smaller in size (257 amino acids compared to 557 in *E. coli*). A secondary structure analysis of the S1 ribosomal protein family revealed that *Tte*-S1 contains analogs of the D1 through D3 domains (59), still including the key elements for binding both 30S subunit and mRNA. S1’s three central domains bind and resolve RNA structures, including that of the Tte preQ1 riboswitch (41), to help accommodate them into the mRNA binding cleft (Figure 5A) (60). S1 loosely binds to the 30S ribosomal subunit and can be depleted during purification (ΔS1-30S) (40) and subsequently be reconstituted by adding recombinant S1 (Methods, Supplementary Figure S12). This approach gives us as tool to further test our protein S1-dependent SD accessibility model.

Our SiM-KARB assay demonstrated that the ΔS1–30S subunit binds to R-mRNA*^+30^* with diminished frequency and stability (Figure 5B); stable binding was recovered only by reconstituting ΔS1–30S with purified S1 (Supplementary Figure S13). Accordingly, the mRNA occupancy histograms showed very low accessibility of individual R-mRNA*^+30^* molecules to ΔS1–30S, counteracted by S1 reconstitution (Figure 5C). Plotting the H, M and L population fractions we found that the L population decreased from 70 ± 8% for ΔS1-30S to 29 ± 4% for WT-30S, while the M and L population increased from 9 ± 2% and 21 ± 7% for ΔS1–30S to 44 ± 3% and 26 ± 5%, respectively, for WT-30S (Figure 5D). These findings suggest that 5′-UTR access is strongly facilitated by S1-mediated RNA unfolding.

Accordingly, we observed a *k_on,slow_* of 0.24 ± 0.02 × 10^6^ M^–1^ s^–1^ for ΔS1-30S, ∼1.4-fold lower than that for WT-30S. In contrast, the *k_off,slow_* of 0.01 ± 0.02 s^–1^ for ΔS1-30S was elevated ∼2-fold over WT-30S (Figure 5D, Supplementary Table S12). That is, not only does S1 unfold the 5′-UTR to facilitate 30S binding (i.e. increases *k_on,slow_*), but it also stabilizes the mRNA-30S complex once formed (i.e. decreases *k_off,slow_*). Stoichiometric (1:1) reconstitution of ΔS1-30S with recombinant S1 recovered particularly the *k_off,slow_* of WT-30S, whereas *k_on,slow_* is restored to a lesser degree (Figure 5D), possibly because free S1 protein in solution coats the pseudoknot, thereby partially blocking 30S recruitment.

These results underscore, first, the important role of S1 protein in accommodating structured RNAs into the 30S subunit's mRNA binding cleft to initiate translation and, second, further support our model that proximal mRNA structure both antagonizes 30S recruitment and accelerates its dissociation.

### Initiation factors only slightly facilitate 30S binding, independent of the 5′-UTR structure

Lastly, we explored how initiation factors (IFs) and initiator fMet-tRNA^fMet^, known to be essential for translation initiation *in vivo*, impact 30S binding in the context of 5′-UTR structure (61,62). While each IF governs a specific function in the initiation process (63,64), we asked to what extent they together influence 30S binding by adding recombinant IF1, IF2, IF3, fMet-tRNA^fMet^ and GTP (0.5 mM) with the 30S into our SiM-KARB assay (Figure 5E) and examining the kinetics of 30S binding to *R-mRNA^+30^*. We found that the fractional 30S occupancy in the presence of IFs and tRNA rose from 11 ± 3% H, 26 ± 2% M and 63 ± 4% L in the absence of preQ_1_ to 1 ± 1% H, 4 ± 2% M, and 95 ± 2% L at saturating preQ_1_, not drastically different from that in the absence of IFs and tRNA (compare Figures 2E and 5F). In the presence of IFs and tRNA^fMet^, *k_on,slow_* increased to 0.39 ± 0.01 × 10^6^ M^–1^ s^–1^ from 0.33 ± 0.04 × 10^6^ M^–1^ s^–1^ in their absence; whereas upon addition of preQ_1_, *k_on,slow_* only slightly increased in the presence of IFs and tRNA^fMet^ to 0.27 ± 0.04 × 10^6^ M^–1^ s^–1^ from 0.24 ± 0.03 × 10^6^ M^–1^ s^–1^ in their absence; finally, *k_off,slow_* remained within error unchanged (Figure 5G and Supplementary Table S13B).

In summary, we thus find that IFs and tRNA^fMet^ slightly increase *k_on,slow_* over the values in their absence, equally in the presence and absence of preQ_1_, while not significantly affecting *k_off,slow_* (Supplementary Figure S14). These observations indicate that the cofactors play a small, supportive role in the earliest stage of translation initiation as probed by 30S binding and exert it equally on the pre-folded apo-riboswitch and the fully folded preQ_1_-bound riboswitch. Similar observations have been made previously, wherein the IFs and tRNA induce only a minor stabilization of the mRNA-30S complex, specifically for mRNAs where translation is initiated by an SD sequence while a cognate start codon is absent (65).

## DISCUSSION

By examining 30S-mRNA interactions at the earliest stage of translation initiation, we reveal here how a small, but strategically placed 5′-UTR structure can influence gene expression. We find that 30S ribosomal subunit binding to the mRNA downstream of an H-type pseudoknotted riboswitch is controlled both by the RNA’s secondary structure, independently of ligand binding to the riboswitch aptamer, and by the occlusion of the SD sequence upon ligand binding. Moreover, preQ_1_ binding itself exerts two distinct effects; first, antagonizing 30S recruitment (i.e. slowing down 30S binding) and, second, destabilizing the 30S-mRNA complex once formed (i.e. accelerating 30S dissociation), giving the mRNA multiple layers of control over translation. This is consistent with the recent study demonstrating the dynamic nature of 30S binding to a structured mRNA and its modulation by preQ_1_ binding to the 5′ riboswitch and some protein factors (34). Mutational analyses further highlight the importance of the SD:anti-SD base pairing to initiate translation (66,67), although it turns out to be only one of several mechanisms by which a translational riboswitch governs gene expression of its hosting mRNA. Such a multi-pronged effect leverages binding of a small ligand into efficient regulation of translation initiation by the much larger bacterial 30S subunit.

Upon decoupling secondary structure and SD sequence, we further find that the precise distance of the aptamer from the SD sequence is a key determinant of initial recruitment of the 30S subunit, with a distance of as small as 4 nt eliminating the aptamer's influence on 30S binding. While the cleft-accommodated mRNA is footprinted from nucleotides (nt) −18 to + 10 on the 30S subunit, exceeding the SD sequence by ∼10 nt on the 5′ side, architecturally the segment of mRNA upstream of the SD sequence is rather solvent exposed (13), rationalizing how steric clashes rapidly diminish on a length scale shorter than the footprint. A similar observation that further supports our interpretation was reported by De Jesus *et al.*, wherein 30S binding to an adenine sensing translational riboswitch is critically dependent on melting of an mRNA structure 5 nt upstream of the SD sequence to release the steric clash between ribosomal proteins and mRNA (45). Together, these observations establish an upper limit for the aptamer-SD distance to allow for effective translational control.

**Figure 6.**
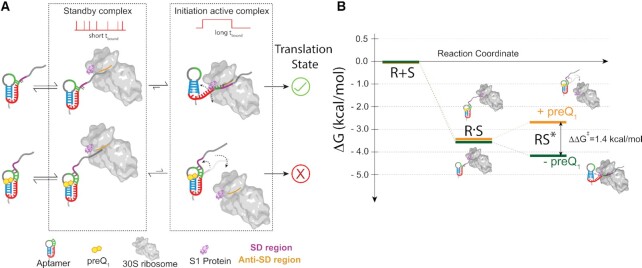
Model for the earliest stage of translation initiation in dependence of an embedded riboswitch structure. (**A**) Independent of the preQ_1_, the 30S dynamically interacts with the R-mRNA scanning for the RBS (and SD sequence) to facilitate translation initiation on the nascent mRNA. Short, transient 30S binding is categorized as standby binding, whereas long binding events represent mRNA accommodation with established SD:anti-SD interactions, required to initiate translation. In the absence of preQ_1_, the SD region is more accessible, favoring complete accommodation of the mRNA through correct SD:anti-SD interactions and more stable 30S binding. By contrast, in the presence of preQ_1_ the SD sequence is partially sequestered, leading to more standby site binding of the mRNA on the 30S subunit and to more frequently disrupted mRNA accommodation, both resulting in unsuccessful translation initiation. (**B**) Free energy diagram calculated from the 30S binding and dissociation rate constants to R-mRNA*^+30^* in the absence or presence of preQ_1_. An energetic penalty of ∼1.4 kcal/mol for formation of the mRNA cleft accommodated complex derives from the dynamic unfolding of the 5′-UTR, necessary for a complete accommodation of the mRNA into the 30S subunit.

Perhaps most strikingly, we find clear evidence for short-lived standby site exploration, sometimes followed by unfolding of the riboswitch for a longer-lived mRNA cleft accommodation, leading to the model in Figure 6A. Consequently, the riboswitch ligand and ribosomal protein S1 counteract in that preQ_1_ tightens the pseudoknot fold while S1 unties it to enhance mRNA cleft accommodation (Figure 6A). We further calculated the unbound dwell times preceding either short (standby) or long (accommodated) bound dwell times (Supplementary Figure S15), and found no systematic difference, consistent with the notion that a standby site bound mRNA can be accommodated and the two processes are likely sequential. Similar S1 dependent engagement of standby-site bound mRNAs has been identified in other bacterial mRNAs where 5′-UTR structures sequester the RBS. For example, Wagner and coworkers showed that ribosomal protein S1 is required for standby-dependent translation in the context of the pseudoknotted 5′-UTR of tisB toxin mRNA (12). Thus, similar to our system, translation requires an initial standby site upon which a 5′-UTR structure is unfolded by S1 on its own or in complex with the 30S subunit for subsequent 30S scanning to the RBS. Based on our kinetic SiM-KARB measurements, we then can further predict a free energy model for the non-equilibrium unfolding of our riboswitch (Figure 6B, Supplementary Figure S16). The initial binding energy into the standby site is very similar in the absence and presence of preQ_1_ (-3.6 kcal/mol). Successive mRNA accommodation by unfolding the mRNA is a spontaneous process yielding –0.5 kcal/mol energy in the absence of preQ_1_, whereas with preQ_1_ bound the 5′-UTR is more stably folded and requires + 0.9 kcal/mol to unfold. The net energetic penalty for cleft-accommodation of the mRNA in the presence of preQ_1_ is thus 1.4 kcal/mol. This small penalty – equivalent to a couple of hydrogen bonds—is a reflection of the exchange of two base pairs in helix P2 of the riboswitch for two base pairs of SD:anti-SD interaction (Figure 1). Previous studies have measured the preQ_1_ mediated aptamer (un)folding kinetics (28,30), which occur on a timescale similar to the 30S binding kinetics reported in the current study. With 30S binding and riboswitch folding taking place on similar time scales, nuanced non-equilibrium kinetic control becomes possible. The multi-pronged effects of the riboswitch on both 30S binding and dissociation are additive so that these seemingly small effects combine for the riboswitch ligand to have significant leverage over translation initiation (Figure 6A).

Our data further suggest that, while individual 30S association and dissociation events each show two rate constants—fast and slow—and hence two bound states, individual mRNA molecules with many binding events also show heterogeneity that can be binned into three categories of overall 30S occupancy over the entire available time window (i.e. L, M and H). The former observation relates to the binding kinetics of the 30S subunit to one less and one more accessible mRNA conformation, whereas the latter represents heterogeneity among individual mRNA molecules. These observations are supported by previous studies suggesting that single mRNAs molecule can retain a differential accessibility over the long term (28), reflecting slow conformational changes of the mRNA possibly due to slow ligand dissociation. In the presence of preQ_1_, R-mRNA accessibility is diminished and in aggregate will lead to significant downregulation of translation.

The leveraging we discovered here of a local, small-scale binding event into the global, large-scale functional process of translation initiation appears to be a recurring theme in RNA biology. For example, in a recent study of a Mn^2+^ sensing riboswitch, a single metal ion binding at the docking core of an RNA four-way junction was shown to alter the global structure to affect transcription (68). Furthermore, highly structured motifs around the RBS of an mRNA often restrict 30S recruitment to control translation efficiency (7,11). The classical model of translation initiation on structured mRNAs invoked an equilibrium thermodynamic process, wherein the 5′-UTR is remodeled into low-energy structures by successive unfolding and refolding that then are accommodated into the mRNA binding cleft of the 30S subunit (69). Recent transcriptome-wide structural studies have refined this model and shown that a non-equilibrium kinetic competition between mRNA unfolding and 30S dissociation governs translation efficiency (7). We demonstrate here that this kinetic competition provides leverage to riboswitches to modulate translation efficiency, even in cases where a small ligand (such as preQ_1_) only provides limited thermodynamic power. Once an mRNA has repelled a 30S subunit, the competition between mRNAs in the bacterial cell dictates that free subunits initiate translation elsewhere, whereas the unoccupied mRNA may fall prey to ribonucleases (33).

In bacteria, translation efficiency as dictated by secondary structure around the RBS plays a significant role in determining the fate of an mRNA (70–72). As initiation complexes form, commitment to translation increases while the vulnerability to mRNA decay decreases, as mediated by the bacterial degradosome, Hfq, and Rho (33,73–75). Consequently, the evolutionary pressure is high to evolve a fine-tuned kinetic modulation of initiation complex formation as a key regulator of global translational activity. The insights presented here will therefore provide an opening for the development of novel antibacterial drugs that derail this finely tuned machinery.

## CODE AVAILABILITY

All custom codes used in this study are available from the corresponding author upon reasonable request.

## DATA AVAILABILITY

The data that support the findings of this study are available from the corresponding author upon reasonable request.

## Supplementary Material

gkac635_Supplemental_File
