# Supplementary material for: Precise tuning of bacterial translation initiation by non-equilibrium 5′-UTR unfolding observed in single mRNAs
Source: Nucleic Acids Res. 2022 Jul 27;50(15):8818–33. doi: 10.1093/nar/gkac635 (PMC9410914; doi:10.1093/nar/gkac635)
Supplement: gkac635_Supplemental_File [file gkac635_supplemental_file.pdf]

Supplementary Information

Precise tuning of bacterial translation initiation by non-equilibrium 5'-UTR unfolding observed in single mRNAs

Sujay Ray†, Shiba S. Dandpat, Surajit Chatterjee and Nils G. Walter*

Single-Molecule Analysis Group, Department of Chemistry, University of Michigan, Ann Arbor, MI 48109, USA. *e-mail: nwalter@umich.edu, † Current address: Wyss Institute for Biologically Inspired Engineering at Harvard University.

Additional Materials and Methods

Global fitting

All the data for association and dissociation rates for each condition were fitted together globally in Origin pro software to get higher accuracy of double-exponential fitting (1,2). To reduce the number of independent parameters associated with double-exponential fitting for both association and dissociation rates as well as considering the heterogeneity of the 30S binding to the R-mRNA^{+30} for each condition, the shorter binding time of the double-exponential fitting was shared across all the conditions of preQ$_1$ and effect of mutations. The global fitting of rates separately yielded two components for each of association ($k_{on,\ slow}$ and $k^{shared}_{on,\ fast}$) and dissociation ($k_{off,\ slow}$ and $k^{shared}_{off,\ fast}$) rates, one of which was variable, and the other was shared over all the conditions. While the shared component of the rate constant (k^{shared}) remained fixed for all conditions, the variable component was observed to be profoundly responsive to the influence of preQ$_1$. The variable rate constant components and their relative contributions were used to compare different conditions to determine the role of preQ$_1$ and strategic mutations on 30S ribosome binding.

Error Analysis:

The errors of the kinetic parameters were estimated by bootstrapping using a Matlab code. Briefly, a data subset is created by chosing fifty molecules at random from the pool of available molecules for a particular experiment. The kinetic parameters are then calculated for those molecules. The process is repeated for ten such ittarations. The standard deviation of those ten subsets is used to represent the error of that measurement.

Distributions of High (H), Mid (M), Low (L) groups of molecules

For 30S binding to R-mRNA^{+30}, three types of 30S binding time distribution were observed by plotting an accumulated distribution of total binding time of 30S for all the conditions pooled together (Supplementary Fig. S7). The cumulative binding time histograms were fitted with three Gaussian plots (with R-Sq value of 0.9914), showing there are three types of total binding populations, which were assigned as H, M, and L to represent the binding

regime they cover. The population H represented the high-range of binding time of the 30S (>30% of the total observation window), M represented mid-range binding time (between 20-30% of the total observation window) and L represented low-range binding time (<10% of the observational window). These regions for H, M, and L were used as cutoffs for calculating cumulative or percentage population for each condition. The cumulative or percentage population for each regime was calculated for each condition by counting the total binning for each regime.

Rastergram distributions

For determining the nature and number of standby (short) and cleft-accommodated (long) binding events, we represented a random selection of 100 molecules for each condition in a rastergram (3,4). First, the molecules were clustered into H, M, and L groups by analyzing the total binding time of the 30S each molecule categorized from the cutoffs estimated from the total binding time histogram distributions shown for each condition. To categorize the binding events into standby (red) and cleft-accommodated (blue) binding events, we took the geometric mean of the two components of binding times obtained from the double-exponential fitting of the cumulative plot for the binding times obtained earlier. We then counted the number of red and blue events for the required conditions and used them to compare any change in the nature of 'standby' and 'cleft-accommodated' binding events under the influence of $preQ_1$ or the effect of strategic mutations. We plotted each individual molecule's 30S binding behavior categorized into the groups of H, M, and L to represent binding events as standby or cleft-accommodated events (shown in red or blue) within each condition. MATLAB scripts for raster plots are available upon request.

Supplementary Figures

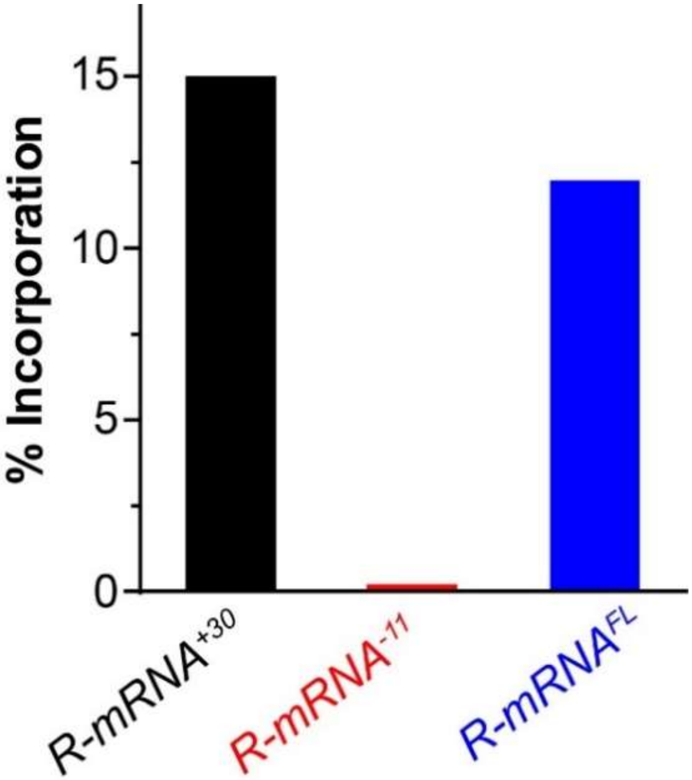

Supplementary Fig. S1| R-mRNA truncations to determine RNA required for 30S IC formation. Comparison of 30S IC formation efficiency on full length R-mRNA^{FL} and R-mRNA^{+30} truncation and R-mRNA^{-11} truncation (no SD/ ORF). The IC was first formed including mRNA, 30S, Initiation factors, ^{32}P-labeled fmet-tRNA and GTP. The reaction was then pelleted by *ultracentrifugation on a sucrose cushion. The ratio of tRNA after and before palleting represents the extent of 30S-IC formation.* As expected, ICs do not form on mRNA that lacks a SD and ORF, whereas efficiency of initiation is similar for full length R-mRNA and R-mRNA^{+30}.

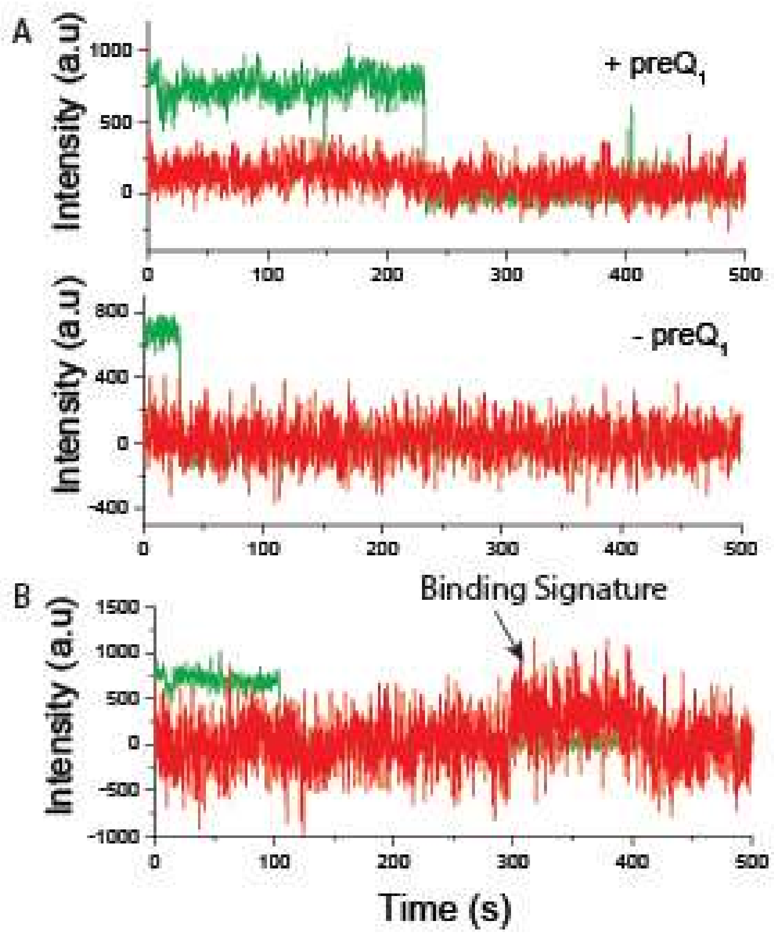

Supplementary Fig. S2|Representative traces for the control experiment using 5'-Cy3 labeled capture DNA in the absence of R-mRNA. (A) Two representative traces showing no Cy5-labeled 30S binding (red). (B) Only one trace showed any evidence for a single Cy5 labeled 30S binding event among more than 100 analyzed traces.

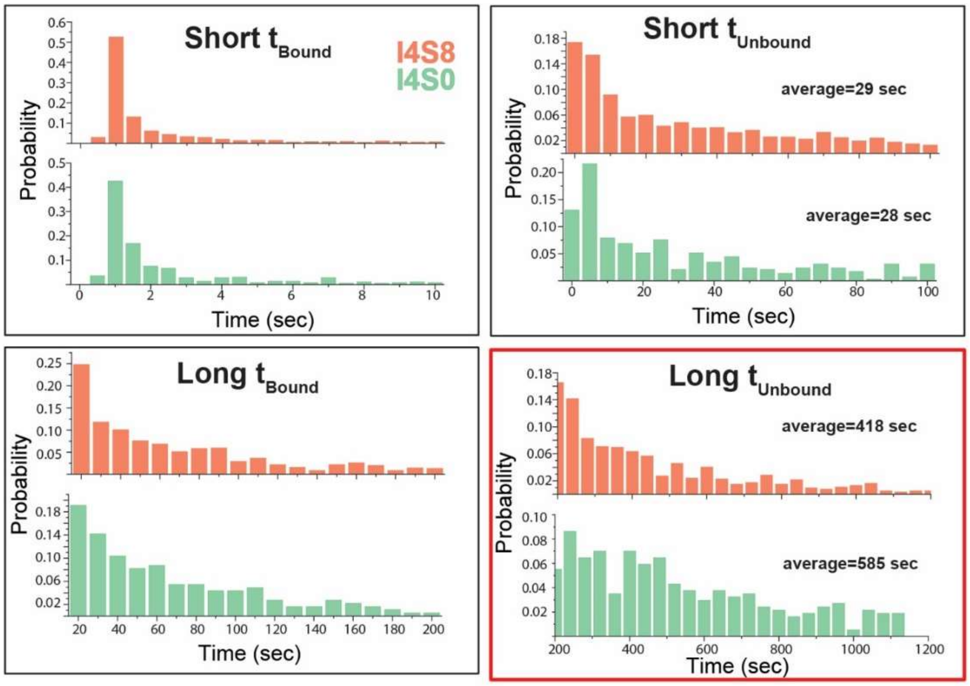

Supplementary Fig. S3| Comparison of histogram for the short and long bound and unbound dwell times for mutants I4S8 and I4S0. Both short and long bound times show virtually identical binding events. While the short unbound times are also similar, however long unbound times are significantly different for the two mutants, indicating that the $k_{on,slow}$ is affected most for the 30S binding.

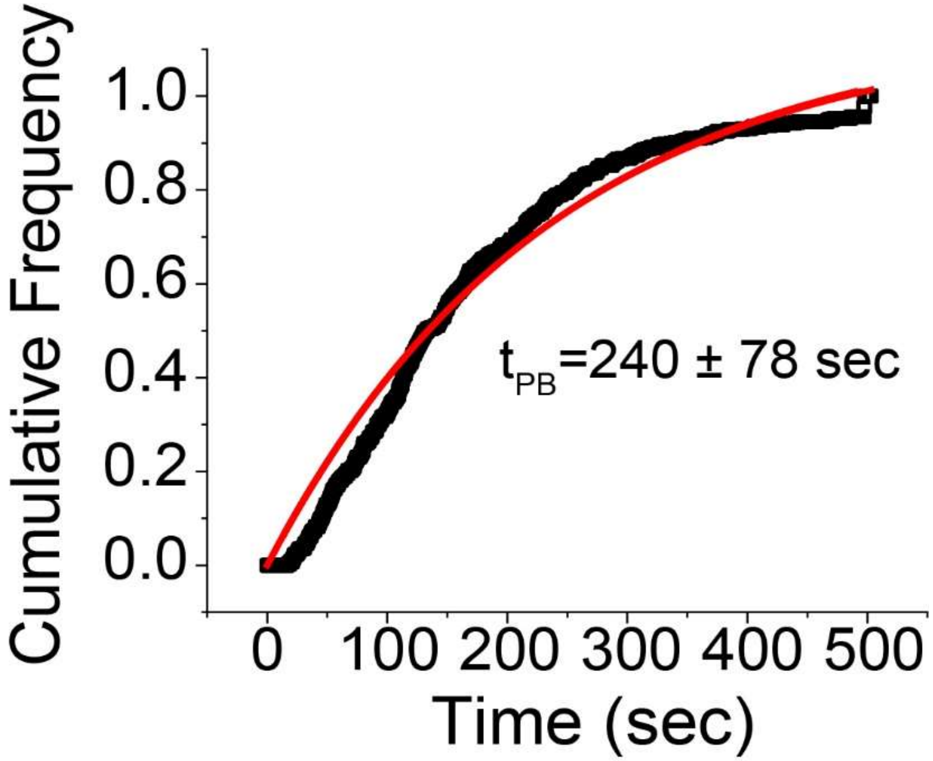

Supplementary Fig. S4| Characterization of photobleaching time for 30S labeling probe. The dual Cy5 labeled 30S labeling-oligo was captured on the surface by a biotinylated capture strand. Same assay conditions and laser intensity and optical parameters were maintained as the SiM-KARB assays. The average characteristic photobleaching time is shown in the graph, which is an estimation of photobleaching of both dye molecules.

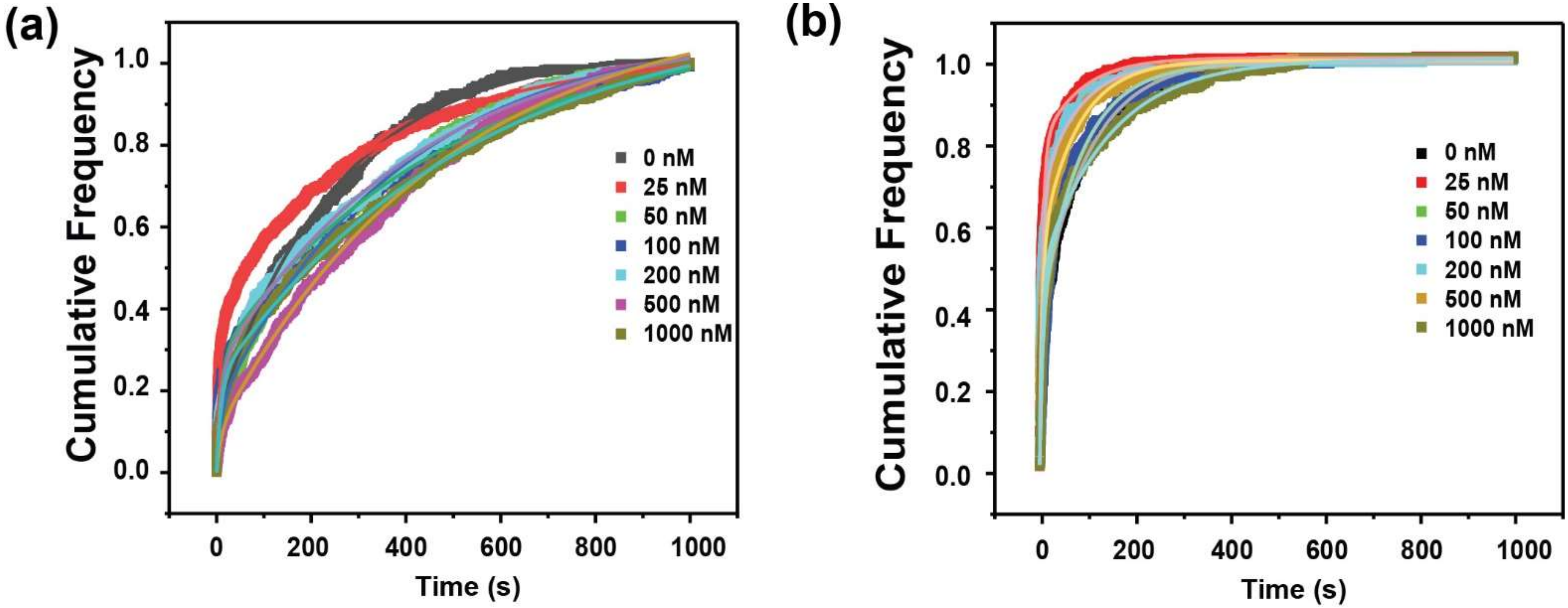

Supplementary Fig. S5| Plots of cumulative unbound (A) and bound (B) dwell times for the 30S binding at different concentrations of preQ$_1$

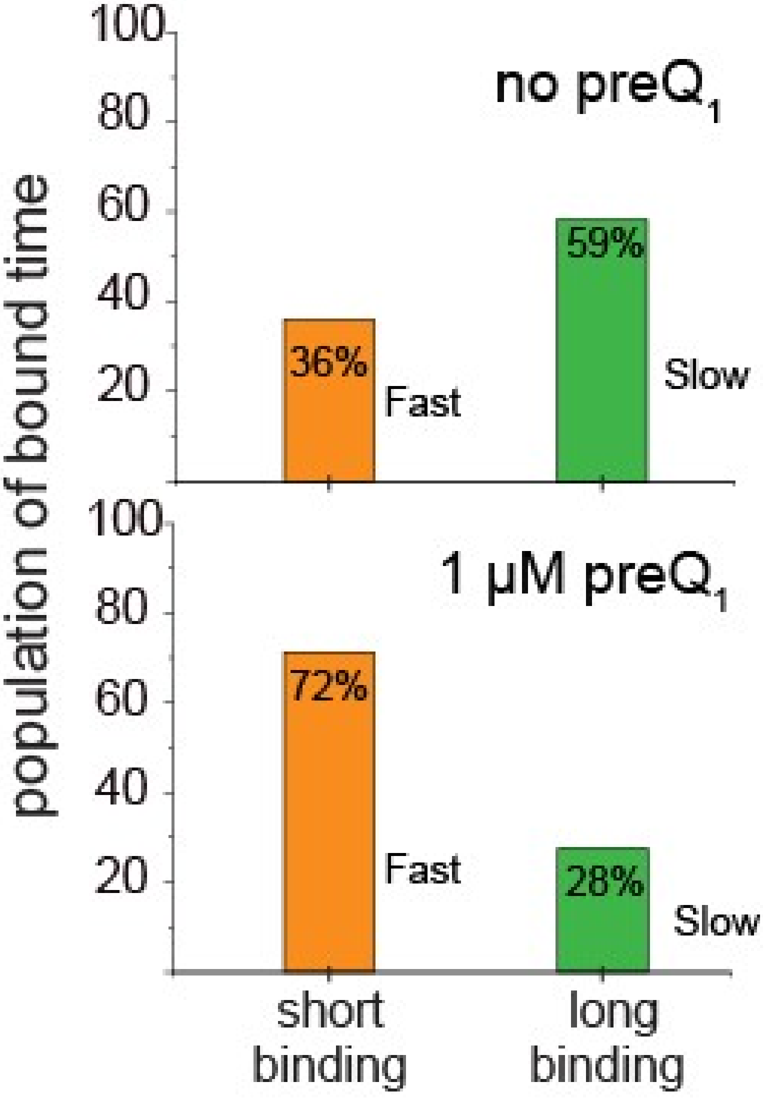

Supplementary Fig. S6|Percentage of long and short binding events for 30S binding to R-mRNA^{+30} in the absence and presence of preQ$_1$ determined from the biexponential fitting of the association ($k_{on,\ slow}$) and dissociation ($k_{off,\ slow}$) rates

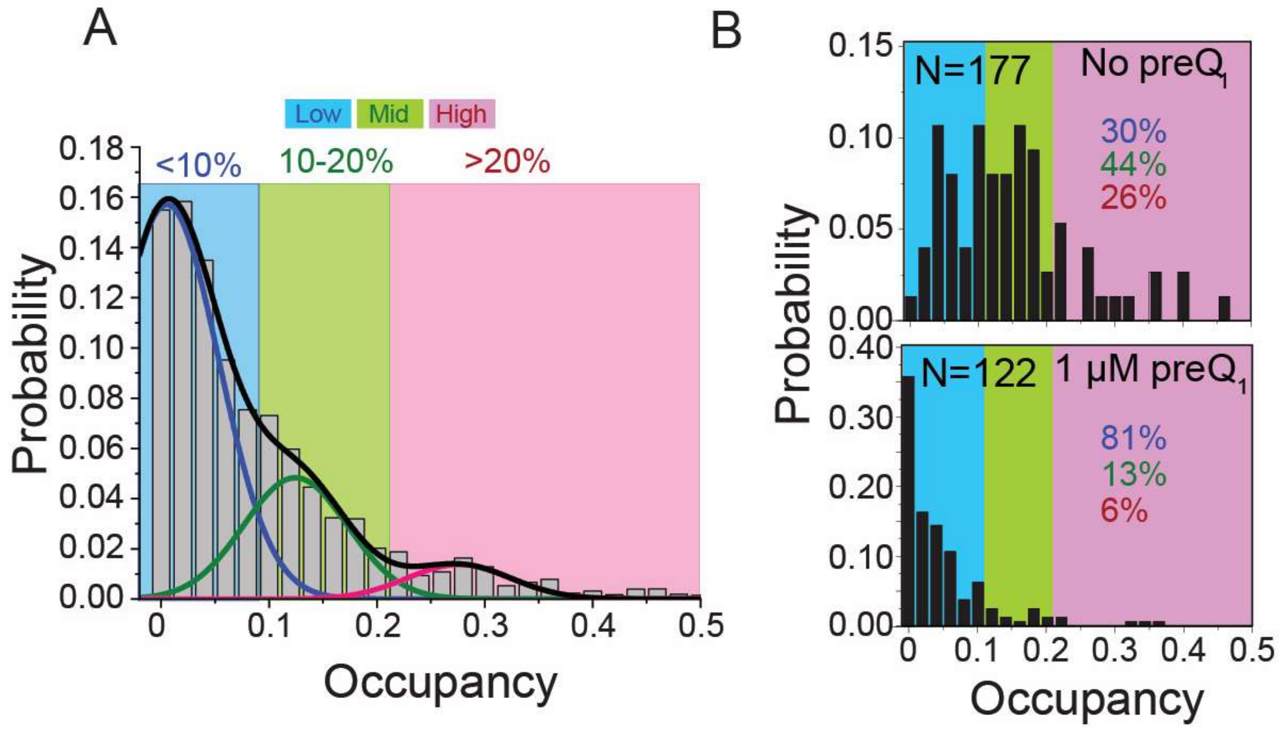

Supplementary Fig. S7| Cumulative plot of all histograms pooled together to identify distribution of bound time populations. (A) For a single mRNA molecule, the total 30S bound time was normalized by the experimental observation window time to calculate the fractional 30S bound time. Based on this fractional 30S bound time, an accessibility ranking was assigned for each mRNA. Molecules where 30S is bound less than 10% (i.e., 0.1 fractional 30S bound time), between 10-20%, and >20% of the total observation time are assigned a low (L), mid (M), and high (H) accessibility ranking, respectively. **(B)** Example of histogram plot for mRNA accessibility.

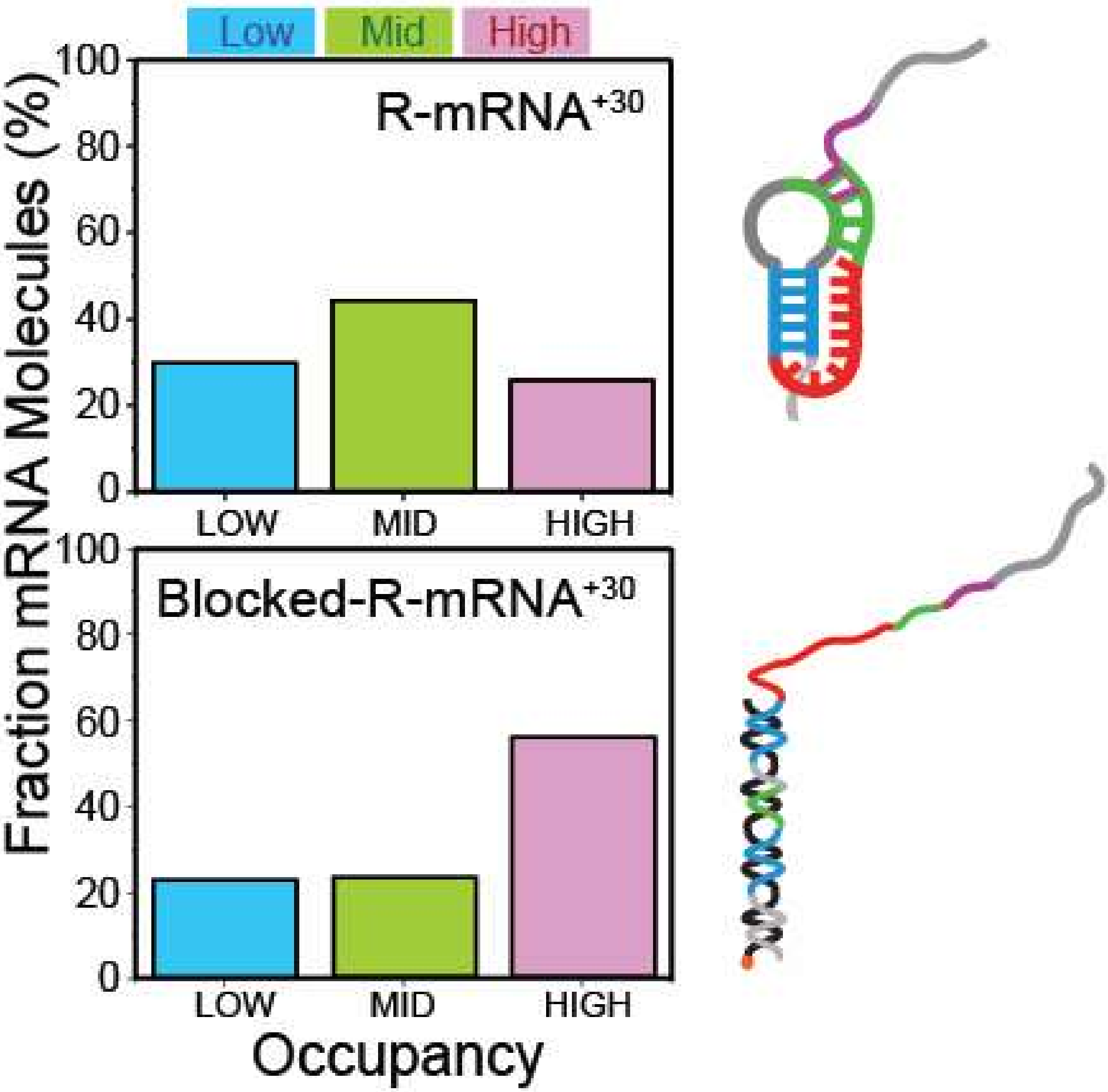

Supplementary Fig. S8|mRNA accessibility ranking of mRNA^{+30} with or without pseudoknot formation. A Blocked-R-mRNA^{+30} construct was formed by extending the capture strand to basepair with the entire P1 stem-loop. Thus, pseudoknot formation (including P2) is blocked. However, the SD-region remains unimpaired. Accordingly, increased 30S binding was observed for this blocked mRNA^{+30}, where 52% of mRNA molecules show high accessibility (lower panel) compared to 26% in the unblocked mRNA^{+30} (upper panel).

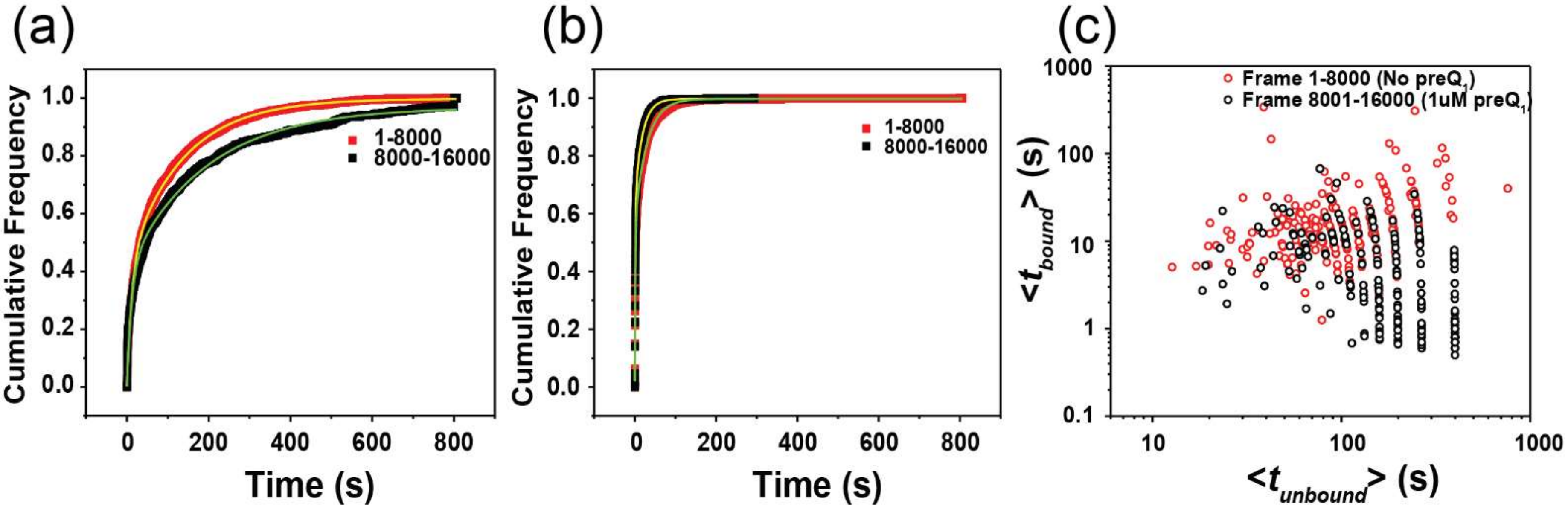

Supplementary Fig. S9| Plots of cumulative unbound and bound dwell times for the 30S binding without and with preQ$_1$ in ligand-jump experiments. (a) Cumulative frequency plot for $t_{unbound}$ of 30S binding to R-mRNA^{+30} without preQ$_1$ (red) before dark period monitored for first 8000 frames and with 1 µM preQ$_1$ (black) after dark period monitored for next 8000 frames. 1 µM preQ$_1$ is added during the dark period that lasts for 5000 frames (1 frame = 0.1 sec) (b) Cumulative frequency plot for t_{bound} of 30S binding to R-mRNA^{+30} without PreQ$_1$ (red) and with 1 µM preQ$_1$ (black) (c) Scatter plot between average unbound time ($t_{unbound}$) vs average bound time (t_{bound}) obtained from the non-equilibrium ligand-jump experiment showing an increase in avg. $t_{unbound}$ and t_{bound}.

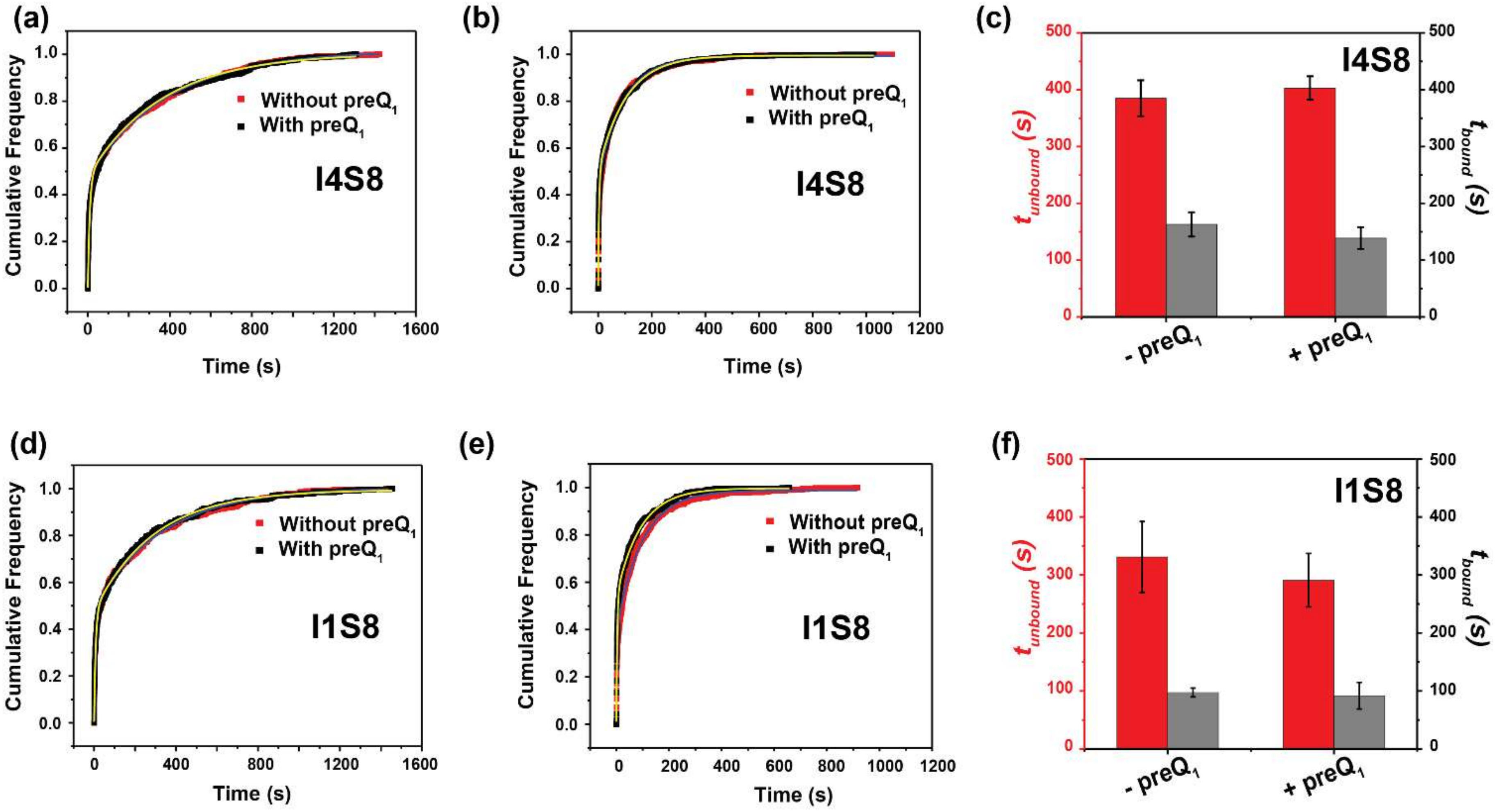

Supplementary Fig. S10| *Effect of preQ$_1$ on mutants I4S8 and I1S8.* (a) Cumulative frequency plot for $t_{unbound}$ for I4S8 in absence and presence of preQ1; (b) Cumulative frequency plot for t_{bound} for I4S8 in the absence and presence of preQ$_1$. (c) Comparison of OFF and ON time for I4S8 without and with preQ$_1$. (d) Cumulative frequency plot for $t_{unbound}$ for I1S8 in the absence and presence of preQ$_1$. (e) Cumulative frequency plot for t_{bound} for I1S8 in the absence or presence of preQ$_1$. (f) Comparison of unbound ($t_{unbound}$, red) and bound (t_{bound}, red) time for I1S8 in the absence and presence of preQ$_1$.

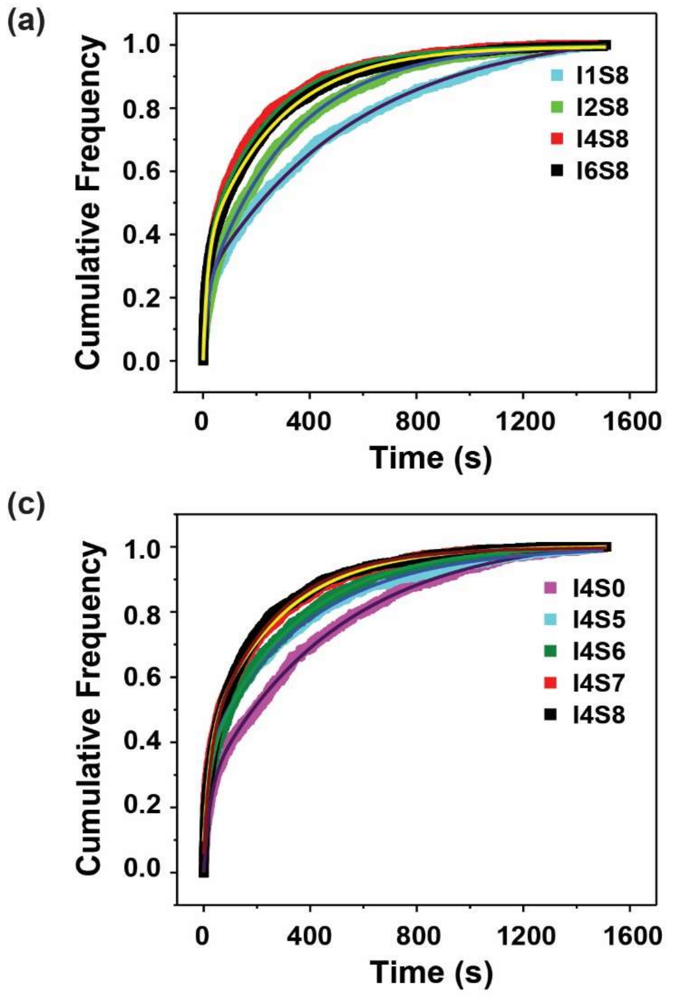

Supplementary Fig. S11| Cumulative plots for bound and unbound times for different mutants. (a) Cumulative frequency plot for $t_{unbound}$ with different SD-aptamer distance (I1S8 to I6S8). (b) Cumulative frequency plot for t_{bound} with different SD-aptamer distance (I1S8 to I6S8). (c) Cumulative frequency plot for $t_{unbound}$ with different length of SD region (I4S8 to I4S0). (b) Cumulative frequency plot for t_{bound} with different SD-length (I4S8 to I4S0).

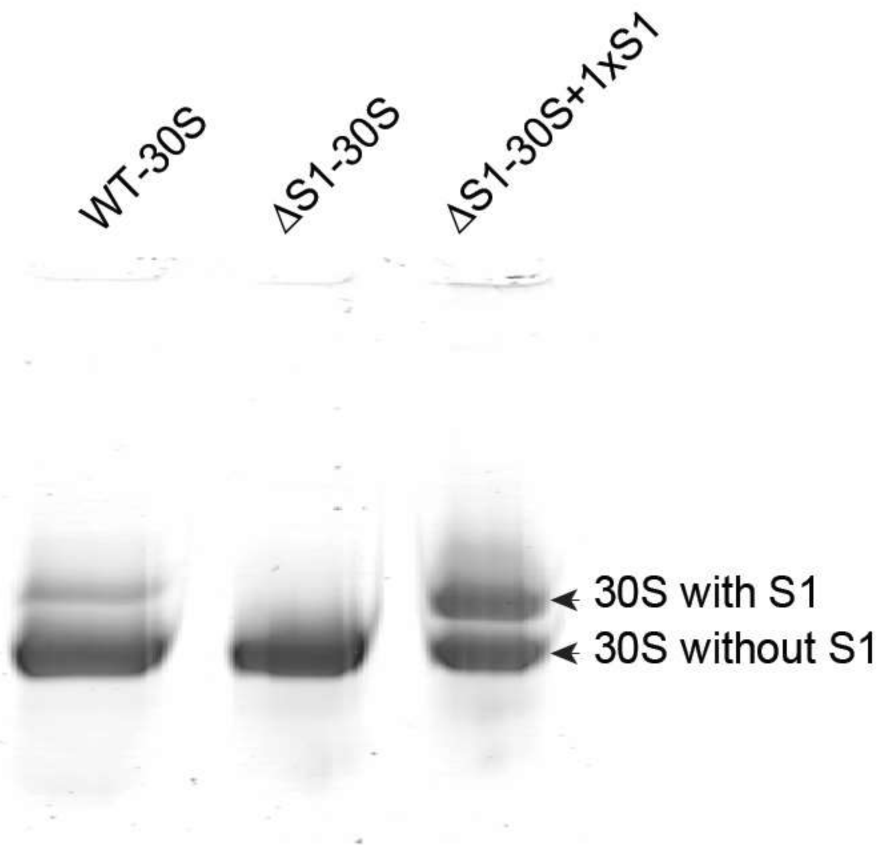

Supplementary Fig. S12| Gel electrophoretic assessment of S1 content in 30S subunits. Salt-washed 30S WT (first lane) shows the presence of 30S with and without S1. S1 was depleted from 30S to form 30S ΔS1 (second lane). S1 was then reconstituted by adding purified S1 (stoichiometric; third lane).

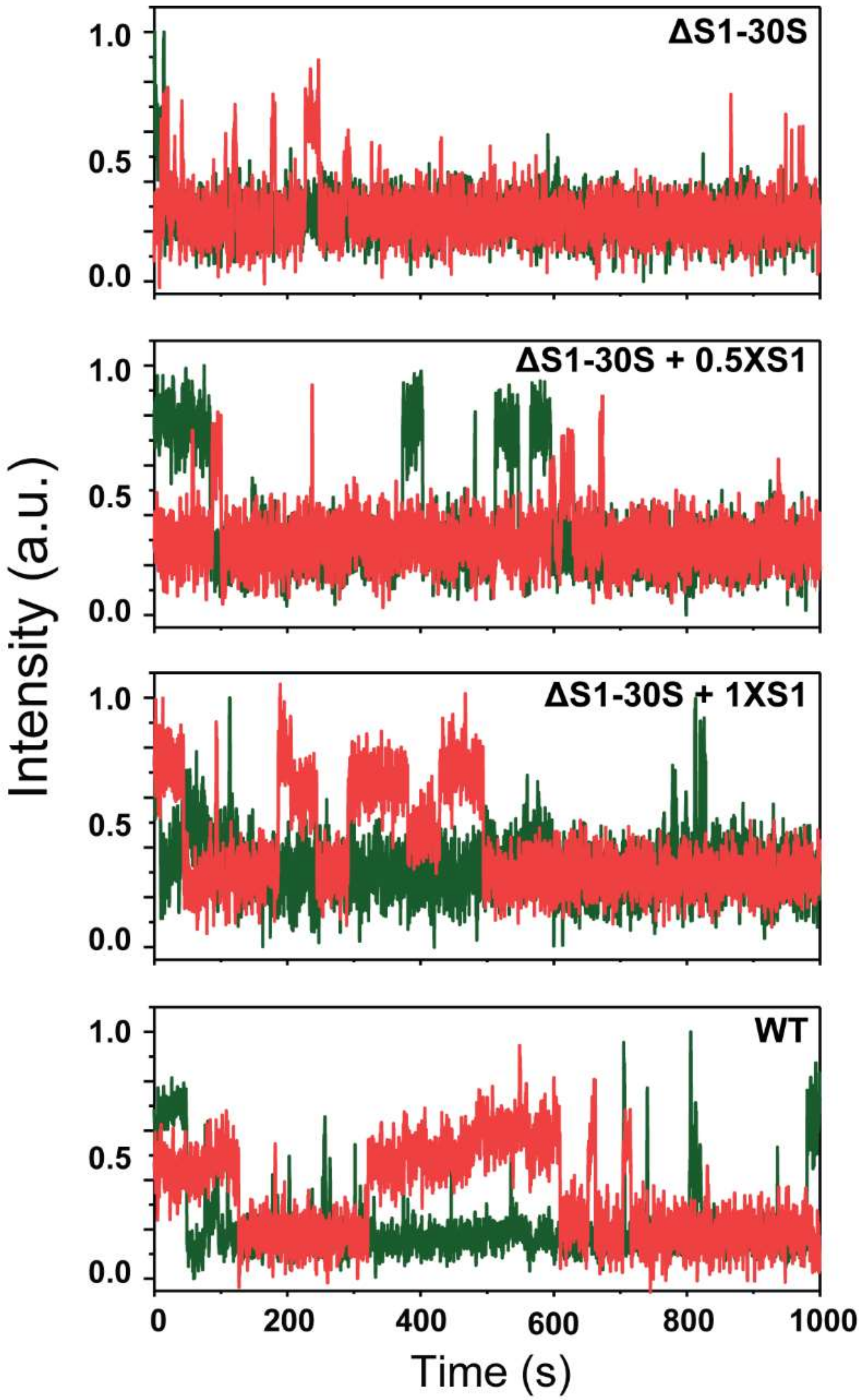

Supplementary Fig. S13| Reconstitution of S1 into 30S subunits. Incorporation of S1 at various molar ratio to ΔS1-30S increased the 30S binding to the R-mRNA^{+30}.

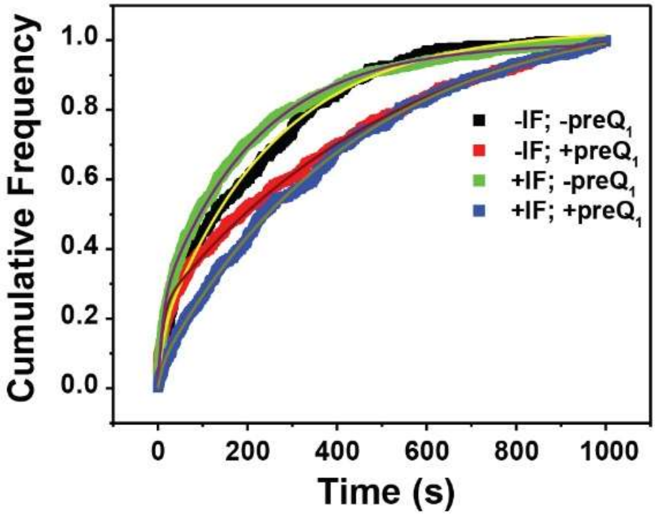

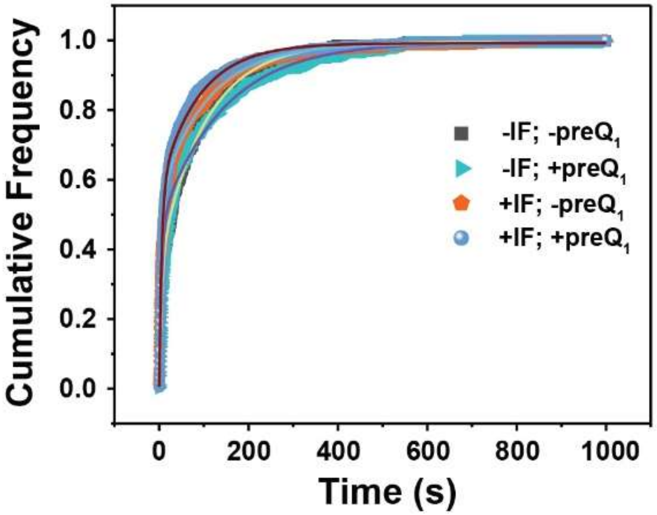

Supplementary Fig. S14| Cumulative plots for unbound times ($t_{unbound}$, left) and bound times (t_{bound}, right) without and with IFs and $preQ_1$.

$$R+S \underset{k_{-1}}{\overset{k_1}{\rightleftharpoons}} R\cdot S \underset{k_{-2}}{\overset{k_2}{\rightleftharpoons}} RS^*$$

$$K_1 = \frac{k_{-1}}{k_1} = \frac{[R][S]}{[R\cdot S]} = e^{\Delta G_{SC}/RT}$$

$$K_2 = \frac{k_{-2}}{k_2} = \frac{[R\cdot S]}{[RS^*]} = e^{\Delta G_{SI}/RT}$$

ΔG_{IA} = binding energy for initiation active complex.

ΔG_{SC} = binding energy for standby complex.

ΔG_{SI} = RNA unfolding energy.

$$\Delta G_{IA} = \Delta G_{SC} + \Delta G_{SI}$$

From our calculations rate values,
we found the energy values in unit of RT,

$\Delta G_{SC}^{-preQ1} = -3.6$	$\Delta G_{SC}^{+preQ1} = -3.6$
$\Delta G_{SI}^{-preQ1} = -0.5$	$\Delta G_{SI}^{+preQ1} = 0.9$
$\Delta G_{IA}^{-preQ1} = -4.1$	$\Delta G_{IA}^{+preQ1} = -2.7$

$$\Delta\Delta G_{\text{unfolding penalty}} = \Delta G_{SI}^{-preQ1} - \Delta G_{SI}^{+preQ1} = 1.4 \text{ kcal/mole}$$

Supplementary Fig. S15| Free Energy estimates of with and without preQ$_1$ conditions.

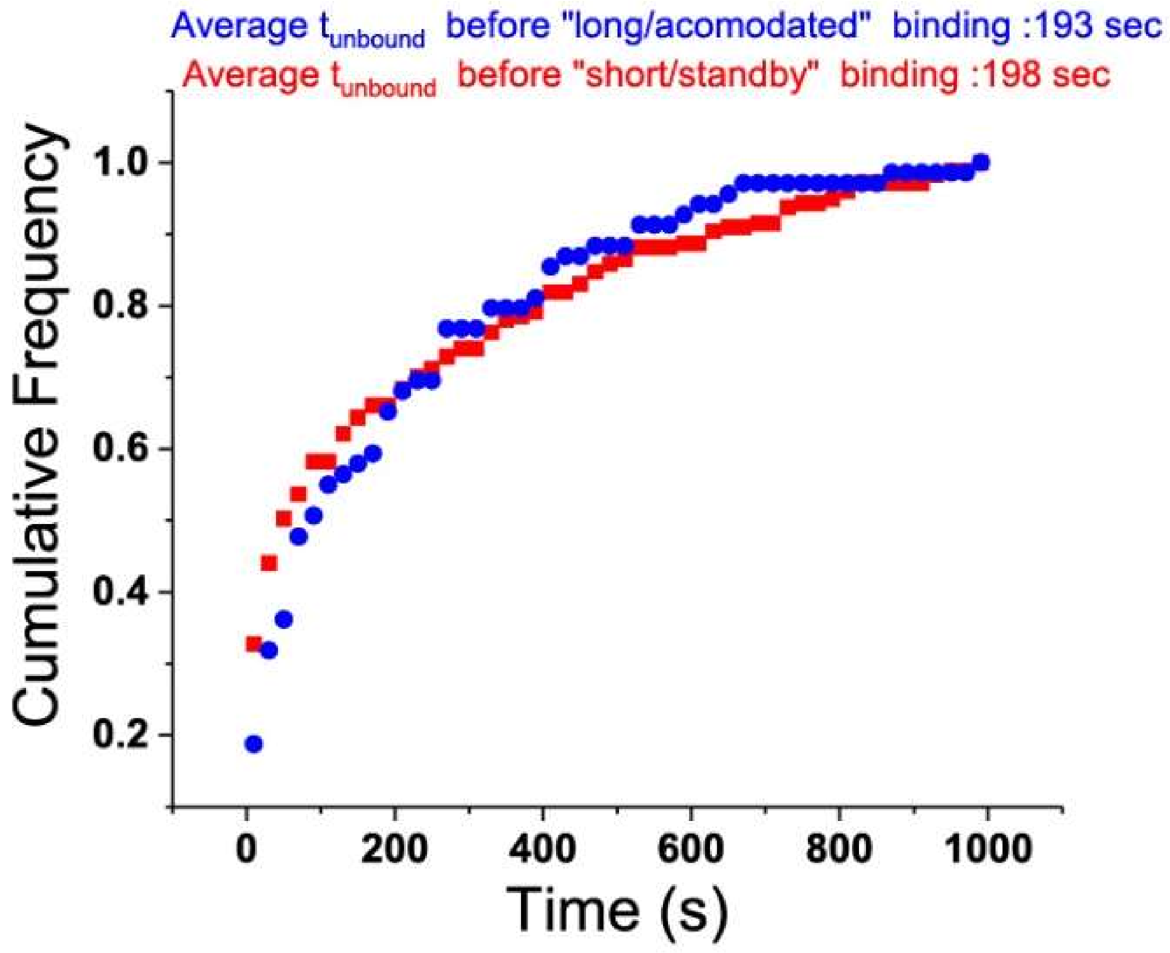

Supplementary Fig. S16| Comparison of average unbound time righ before a long or accommodated binding (in blue) and right before a short/standby binding (in red). Practically both unbound times (and hence on rates) are similar, suggesting evidence of standby binding followed by long accommodated binding.

Supplementary Tables

Supplementary Table S1. *List of RNA sequence used for microscopy and biochemistry. The surface captured part of the mRNA is shown in gray, the aptamer is italized, the SD sequence is non-italizised and underlined, an insertion is bolded and highlighted in gray, the start codon is italized and green.*

Name	Sequence
WT R-mRNA^{+30} (I-2S8)	GGGCAGUGAGCAACAAAAUGC*UCACCUGGGUCGCAGUAACCCCAGUUAACA AAACA*AGGGAGGU*AAUUUUGUG*CCCAAAAAAAGAAUAAAAGAUUUAGCU
I1S8 R-mRNA	GGGCAGUGAGCAACAAAAUGC*UCACCUGGGUCGCAGUAACCCCAGUUAACA AAACAAG***U**AGGGAGGU*AAUUUUGUG*CCCAAAAAAAGAAUAAAAGAUUUAG CU
I2S8 R-mRNA	GGGCAGUGAGCAACAAAAUGC*UCACCUGGGUCGCAGUAACCCCAGUUAACA AAACAAG***AU**AGGGAGGU*AAUUUUGUG*CCCAAAAAAAGAAUAAAAGAUUUA GCU
I4S8 R-mRNA	GGGCAGUGAGCAACAAAAUGC*UCACCUGGGUCGCAGUAACCCCAGUUAACA AAACAAG***AUAU**AGGGAGGU*AAUUUUGUG*CCCAAAAAAAGAAUAAAAGAUU UAGCU
I6S8 R-mRNA	GGGCAGUGAGCAACAAAAUGC*UCACCUGGGUCGCAGUAACCCCAGUUAACA AAACAAG***AUAUAU**AGGGAGGU*AAUUUUGUG*CCCAAAAAAAGAAUAAAAGA UUUAGCU
I4S7 R-mRNA	GGGCAGUGAGCAACAAAAUGC*UCACCUGGGUCGCAGUAACCCCAGUUAACA AAACAAG***AUAU**UGGGAGGU*AAUUUUGUG*CCCAAAAAAAGAAUAAAAGAUU UAGCU
I4S6 R-mRNA	GGGCAGUGAGCAACAAAAUGC*UCACCUGGGUCGCAGUAACCCCAGUUAACA AAACAAG***AUAU**UUGGAGGU*AAUUUUGUG*CCCAAAAAAAGAAUAAAAGAUU UAGCU
I4S5 R-mRNA	GGGCAGUGAGCAACAAAAUGC*UCACCUGGGUCGCAGUAACCCCAGUUAACA AAACAAG***AUAU**UUUGAGGU*AAUUUUGUG*CCCAAAAAAAGAAUAAAAGAUU UAGCU
I4S0 R-mRNA	GGGCAGUGAGCAACAAAAUGC*UCACCUGGGUCGCAGUAACCCCAGUUAACA AAACAAG***AUAU***AUAUAUAUAAUUUUGUG*CCCAAAAAAAGAAUAAAAGAUU UAGCU
Capture Strand DNA	*5'-GCATTTTGTTGCTCACTGCCC-biotin-3'*
Capture Strand DNA with Cy3	*5'-Cy3-GCATTTTGTTGCTCACTGCCC-biotin-3'*
30S DNA labeling probe	*5' – Cy5- GGG AGA TCA GGA TA -Cy5 3'*

Supplementary ***Table S2.*** *Table of site-directed mutagenesis primers used to generate mutant aptamer series of plasmids*

Primer Sample	Primer Sequence (5' to 3')
Tte_Forward primer	/5Phos/ GAGGTAATTTTGTGCCC
Tte_I1S8 Reverse Primer	/5Phos/ CCTACTTGTTTTGTTAACTGG
Tte_I2S8 Reverse Primer	/5Phos/ CCTATCTTGTTTTGTTAACTGG
Tte_I4S8 Reverse Primer	/5Phos/ CCTATATCTTGTTTTGTTAACTGG
Tte_I6S8 Reverse Primer	/5Phos/ CCTATATATCTTGTTTTGTTAACTGG
Tte_I4S7 Reverse Primer	/5Phos/ CCAATATCTTGTTTTGTTAACTGG
Tte_I4S6 Reverse Primer	/5Phos/ CAAATATCTTGTTTTGTTAACTGG
Tte_I4S5 Forward Primer	/5Phos/ CCAAAAAAAGAATAAAAGATTTAGC
Tte_I4S5 Reverse Primer	/5Phos/ GCACAAAATTACCTCAAAATATCTTG
Tte_I4S0 Reverse Primer	/5Phos/ GCACAAAATTATATATATATATATCTTGTTTTGTTAAC

Supplementary Table S3 A *List of all unbound times ($t_{unbound}$) and association rates (k_{on}) for 30S binding to R-mRNA^{+30} at different preQ$_1$ concentration.*

[PreQ$_1$] (nM)	$t_{unbound,slow}$ (s)	±Δ $t_{unbound,slow}$ (s)	A_1	±ΔA_1	$k_{on,slow}$ (×10^{6}M^{-1}s^{-1})	±Δ $k_{on,slow}$ (×10^{6}M^{-1}s^{-1})	$k_{on,slow}$ (PB corrected) (×10^{6}M^{-1}s^{-1})	±Δ $k_{on,slow}$ (PB corrected) (×10^{6}M^{-1}s^{-1})
0	299.9	35.3	0.89	0.04	0.17	0.02	0.33	0.04
50	410.8	19.3	0.93	0.03	0.12	0.01	0.28	0.01
100	424.2	38.8	0.83	0.05	0.12	0.01	0.27	0.03
200	490.5	92.8	0.85	0.05	0.10	0.02	0.26	0.05
500	539.9	36.8	0.85	0.05	0.09	0.01	0.25	0.02
1000	607.5	86.1	0.96	0.08	0.08	0.01	0.24	0.03

[PreQ$_1$] (nM)	$t_{unbound,fast}^{shared}$ (s)	±Δ $t_{unbound,fast}^{shared}$ (s)	A_2	±ΔA_2	$k_{on,fast}$ (×10^{6}M^{-1}s^{-1})	±Δ $k_{on,fast}$ (×10^{6}M^{-1}s^{-1})	$k_{on,fast}$ (PB corrected) (×10^{6}M^{-1}s^{-1})	±Δ $k_{on,fast}$ (PB corrected) (×10^{6}M^{-1}s^{-1})
0	10.3	1.38	0.15	0.03	4.85	0.65	4.84	0.65
50	10.3	1.38	0.15	0.02	4.85	0.65	4.84	0.65
100	10.3	1.38	0.29	0.04	4.85	0.65	4.84	0.65
200	10.3	1.38	0.24	0.03	4.85	0.65	4.84	0.65
500	10.3	1.38	0.10	0.01	4.85	0.65	4.84	0.65
1000	10.3	1.38	0.22	0.01	4.85	0.65	4.84	0.65

Supplementary Table S3 B *List of all bound times (t_{bound}) and dissociation rates (k_{off}) for 30S binding to R-mRNA^{+30} at different preQ$_1$ concentration.*

[PreQ$_1$] (nM)	$t_{bound,slow}$ (s)	±Δ $t_{bound,slow}$ (s)	A_1	±ΔA_1	$k_{off,slow}$ (s^{-1})	±Δ $k_{off,slow}$ (s^{-1})	$k_{off,slow}$ (PB Corrected) (s^{-1})	±Δ$k_{off,slow}$ (PB Corrected) (s^{-1})
0	137.2	41.3	0.58	0.08	0.007	0.002	0.002	0.001
50	110.8	29.9	0.48	0.07	0.009	0.002	0.004	0.001
100	96.2	58.9	0.41	0.03	0.012	0.002	0.005	0.003
200	100.1	16.3	0.48	0.06	0.010	0.002	0.005	0.001
500	90.7	18.5	0.49	0.01	0.007	0.001	0.006	0.001
1000	86.1	13.8	0.28	0.08	0.008	0.004	0.006	0.001

[PreQ$_1$] (nM)	$t_{bound,fast}^{shared}$ (s)	±Δ $t_{bound,fast}^{shared}$ (s)	A_2	±ΔA_2	$k_{off,fast}$ (s^{-1})	±Δ $k_{off,fast}$ (s^{-1})	$k_{off,fast}$ (PB Corrected) (s^{-1})	±Δ$k_{off,fast}$ (PB Corrected) (s^{-1})
0	7.2	1.10	0.36	0.03	0.138	0.021	0.133	0.020
50	7.2	1.10	0.51	0.07	0.138	0.021	0.133	0.020
100	7.2	1.10	0.58	0.08	0.138	0.021	0.133	0.020
200	7.2	1.10	0.51	0.07	0.138	0.021	0.133	0.020
500	7.2	1.10	0.38	0.10	0.138	0.021	0.133	0.020
1000	7.2	1.10	0.72	0.03	0.138	0.021	0.133	0.020

Supplementary Table S4 *Counts of short (represented in red) and long (represented in blue) binding events from the raster plot of 100 molecules in the absence and presence of preQ$_1$ added to R-mRNA^{+30}*

	**Without PreQ$_1$**			**With PreQ$_1$**		
Total Molecules	*# of short binding*	*# of long binding*	*Total binding*	*# of short binding*	*# of long binding*	*Total binding*
100	*194*	*97*	*291*	*184*	*65*	*248*

Supplementary Table S5 A Ligand Jump Experiments. *Transition of accessibility with and without preQ$_1$*

**Without PreQ$_1$ (Before ligand flow)**			**With PreQ$_1$ (After ligand flow)**		
			Low	*Mid*	*High*
Low	*102 (45%)*	Transition →	*90*	*11*	*1*
Mid	*70 (31%)*		*62*	*6*	*2*
High	*55 (24%)*		*35*	*11*	*9*
			187 (82%)	*28 (12%)*	*12 (6%)*
46% molecules stay in the same rank (Low → Low, Mid → Mid, High → High).<br>48% molecules have reduced accessibility rank (Mid → Low, High → Low/Mid).<br>6% molecules have increased accessibility rank (Low →Mid/ High, Mid → High).					
15% molecule transition to completely inaccessible 30S binding once preQ$_1$ is introduced.					

Supplementary Table S5 B Ligand Jump Experiments. *Counts of short (represented in red) and long (represented in blue) binding events from the raster plot of 100 molecules in the absence and presence of preQ$_1$ to R-mRNA^{+30}*

	**Without PreQ$_1$ (Before ligand flow)**				**With PreQ$_1$ (After ligand flow)**			
	# of total molecules	*# of short binding*	*# of long binding*	*Total binding*	*# of total molecules*	*# of short binding*	*# of long binding*	*Total binding*
Total	*100*	*506*	*254*	*760*	*100*	*356*	*107*	*463*

Supplementary Table S6 A *List of all unbound times ($t_{unbound}$) and association rates (k_{on}) for mutants with different SD-aptamer distance (I1S8 to I6S8)*

	$t_{unbound,slow}$ (s)	±Δ $t_{unbound,slow}$ (s)	A_1	±ΔA_1	$k_{on,slow}$ (×$10^6 M^{-1}s^{-1}$)	±Δ $k_{on,slow}$ (×$10^6 M^{-1}s^{-1}$)	$k_{on,slow}$ (PB corrected) (×$10^6 M^{-1}s^{-1}$)	±Δ $k_{on,slow}$ (PB corrected) (×$10^6 M^{-1}s^{-1}$)
**I1S8**	558.7	55.6	0.79	0.02	0.089	0.009	0.248	0.025
**I2S8**	327.4	11.2	0.79	0.02	0.153	0.005	0.311	0.011
**I4S8**	247.3	9.4	0.62	0.01	0.202	0.008	0.361	0.014
**I6S8**	277.7	10.6	0.64	0.01	0.180	0.007	0.338	0.013

	$t^{shared}_{unbound,fast}$ (s)	±Δ $t^{shared}_{unbound,fast}$ (s)	A_2	±ΔA_2	$k_{on,fast}$ (×$10^6 M^{-1}s^{-1}$)	±Δ $k_{on,fast}$ (×$10^6 M^{-1}s^{-1}$)	$k_{on,fast}$ (PB corrected) (×$10^6 M^{-1}s^{-1}$)	±Δ $k_{on,fast}$ (PB corrected) (×$10^6 M^{-1}s^{-1}$)
**I1S8**	16.5	1.53	0.25	0.01	3.034	0.283	3.189	0.296
**I2S8**	16.5	1.53	0.21	0.01	3.034	0.283	3.189	0.296
**I4S8**	16.5	1.53	0.37	0.01	3.034	0.283	3.189	0.296
**I6S8**	16.5	1.53	0.35	0.01	3.034	0.283	3.189	0.296

Supplementary Table S6 B *List of all bound times (t_{bound}) and dissociation rates (k_{off}) for mutants with different SD-aptamer distance (I1S8 to I6S8)*

	$t_{bound,slow}$ (s)	±Δ $t_{bound,slow}$ (s)	A_1	±ΔA_1	$k_{off,slow}$ (×$10^6 M^{-1}s^{-1}$)	±Δ $k_{off,slow}$ (×$10^6 M^{-1}s^{-1}$)	$k_{off,slow}$ (PB corrected) (×$10^6 M^{-1}s^{-1}$)	±Δ $k_{off,slow}$ (PB corrected) (×$10^6 M^{-1}s^{-1}$)
**I1S8**	84.8	20.2	0.48	0.02	0.012	0.003	0.007	0.002
**I2S8**	58.2	8.0	0.33	0.03	0.017	0.002	0.012	0.002
**I4S8**	99.3	37.5	0.30	0.01	0.010	0.004	0.005	0.002
**I6S8**	74.5	42.8	0.19	0.07	0.013	0.008	0.008	0.005

	$t^{shared}_{bound,fast}$ (s)	±Δ $t^{shared}_{bound,fast}$ (s)	A_2	±ΔA_2	$k_{off,fast}$ (×$10^6 M^{-1}s^{-1}$)	±Δ $k_{off,fast}$ (×$10^6 M^{-1}s^{-1}$)	$k_{off,fast}$ (PB corrected) (×$10^6 M^{-1}s^{-1}$)	±Δ $k_{off,fast}$ (PB corrected) (×$10^6 M^{-1}s^{-1}$)
**I1S8**	2.3	0.85	0.52	0.02	0.435	0.162	0.429	0.159
**I2S8**	2.3	0.85	0.66	0.03	0.435	0.162	0.429	0.159
**I4S8**	2.3	0.85	0.70	0.01	0.435	0.162	0.429	0.159
**I6S8**	2.3	0.85	0.81	0.07	0.435	0.162	0.429	0.159

Supplementary Table S7 A *Unbound times (t_{bound}) and association rates (k_{on}) for the influence of $preQ_1$ on mutant I1S8*

	**$t_{unbound,slow}$ (s)**	**±Δ $t_{unbound,slow}$ (s)**	**A_1**	**±ΔA_1**	**$k_{on,slow}$ ($\times 10^6 M^{-1}s^{-1}$)**	**±Δ $k_{on,slow}$ ($\times 10^6 M^{-1}s^{-1}$)**	**$k_{on,slow}$ (PB corrected) ($\times 10^6 M^{-1}s^{-1}$)**	**±Δ $k_{on,slow}$ (PB corrected) ($\times 10^6 M^{-1}s^{-1}$)**
I1S8 -$preQ_1$	*330.9*	*61.0*	*0.51*	*0.03*	*0.151*	*0.028*	*0.309*	*0.057*
I1S8 +$preQ_1$	*290.9*	*46.1*	*0.52*	*0.01*	*0.172*	*0.027*	*0.330*	*0.052*

	**$t^{shared}_{unbnd,fast}$ (s)**	**±Δ $t^{shared}_{unbound,fast}$ (s)**	**A_2**	**±ΔA_2**	**$k_{on,fast}$ ($\times 10^6 M^{-1}s^{-1}$)**	**±Δ $k_{on,fast}$ ($\times 10^6 M^{-1}s^{-1}$)**	**$k_{on,fast}$ (PB corrected) ($\times 10^6 M^{-1}s^{-1}$)**	**±Δ $k_{on,fast}$ (PB corrected) ($\times 10^6 M^{-1}s^{-1}$)**
I1S8 -$preQ_1$	*7.8*	*1.63*	*0.49*	*0.03*	*6.389*	*1.331*	*6.569*	*1.373*
I1S8 +$preQ_1$	*7.8*	*1.63*	*0.48*	*0.01*	*6.389*	*1.331*	*6.569*	*1.373*

Supplementary Table S7 B *Bound times (t_{bound}) and dissociation rates (k_{off}) for the influence of $preQ_1$ on mutant I1S8*

	**$t_{bound,slow}$ (s)**	**±Δ $t_{bound,slow}$ (s)**	**A_1**	**±ΔA_1**	**$k_{off,slow}$ ($\times 10^6 M^{-1}s^{-1}$)**	**±Δ $k_{off,slow}$ ($\times 10^6 M^{-1}s^{-1}$)**	**$k_{off,slow}$ (PB corrected) ($\times 10^6 M^{-1}s^{-1}$)**	**±Δ $k_{off,slow}$ (PB corrected) ($\times 10^6 M^{-1}s^{-1}$)**
I1S8 -$preQ_1$	*168.3*	*7.5*	*0.59*	*0.02*	*0.006*	*0.001*	*0.001*	*0.001*
I1S8 +$preQ_1$	*219.0*	*23.0*	*0.46*	*0.01*	*0.005*	*0.001*	*0.001*	*0.001*

	**$t^{shared}_{bound,fast}$ (s)**	**±Δ $t^{shared}_{bound,fast}$ (s)**	**A_2**	**±ΔA_2**	**$k_{off,fast}$ ($\times 10^6 M^{-1}s^{-1}$)**	**±Δ $k_{off,fast}$ ($\times 10^6 M^{-1}s^{-1}$)**	**$k_{off,fast}$ (PB corrected) ($\times 10^6 M^{-1}s^{-1}$)**	**±Δ $k_{off,fast}$ (PB corrected) ($\times 10^6 M^{-1}s^{-1}$)**
I1S8 -$preQ_1$	*5.8*	*0.51*	*0.39*	*0.02*	*0.172*	*0.015*	*0.167*	*0.015*
I1S8 +$preQ_1$	*5.8*	*0.51*	*0.53*	*0.01*	*0.172*	*0.015*	*0.167*	*0.015*

Supplementary Table S8 A *Unbound times ($t_{unbound}$) and association rates (k_{on}) for the influence of $preQ_1$ on mutant construct I4S8*

	$t_{unbound,slow}$ (s)	±Δ $t_{unbound,slow}$ (s)	A_1	±ΔA_1	$k_{on,slow}$ ($\times 10^6 M^{-1}s^{-1}$)	±Δ $k_{on,slow}$ ($\times 10^6 M^{-1}s^{-1}$)	$k_{on,slow}$ (PB corrected) ($\times 10^6 M^{-1}s^{-1}$)	±Δ $k_{on,slow}$ (PB corrected) ($\times 10^6 M^{-1}s^{-1}$)
I4S8 -$preQ_1$	385.0	31.4	0.54	0.01	0.129	0.011	0.288	0.024
I4S8 +$preQ_1$	403.0	20.7	0.53	0.02	0.124	0.006	0.282	0.015

	$t_{unbnd,fast}^{shared}$ (s)	±Δ $t_{unbound,fast}^{shared}$ (s)	A_2	±ΔA_2	$k_{on,fast}$ ($\times 10^6 M^{-1}s^{-1}$)	±Δ $k_{on,fast}$ ($\times 10^6 M^{-1}s^{-1}$)	$k_{on,fast}$ (PB corrected) ($\times 10^6 M^{-1}s^{-1}$)	±Δ $k_{on,fast}$ (PB corrected) ($\times 10^6 M^{-1}s^{-1}$)
I4S8 -$preQ_1$	9.6	1.6	0.45	0.018	5.192	0.869	5.367	0.894
I4S8 +$preQ_1$	9.6	1.6	0.48	0.020	5.192	0.869	5.367	0.894

Supplementary Table S8 B *Bound times (t_{bound}) and dissociation rates (k_{off}) for the influence of $preQ_1$ on mutant construct I4S8*

	$t_{bound,slow}$ (s)	±Δ $t_{bound,slow}$ (s)	A_1	±ΔA_1	$k_{off,slow}$ ($\times 10^6 M^{-1}s^{-1}$)	±Δ $k_{off,slow}$ ($\times 10^6 M^{-1}s^{-1}$)	$k_{off,slow}$ (PB corrected) ($\times 10^6 M^{-1}s^{-1}$)	±Δ $k_{off,slow}$ (PB corrected) ($\times 10^6 M^{-1}s^{-1}$)
I4S8 -$preQ_1$	162.9	21.5	0.51	0.03	0.006	0.001	0.001	0.0001
I4S8 +$preQ_1$	138.9	19.1	0.52	0.02	0.007	0.001	0.002	0.0003

	$t_{bound,fast}^{shared}$ (s)	±Δ $t_{bound,fast}^{shared}$ (s)	A_2	±ΔA_2	$k_{off,fast}$ ($\times 10^6 M^{-1}s^{-1}$)	±Δ $k_{off,fast}$ ($\times 10^6 M^{-1}s^{-1}$)	$k_{off,fast}$ (PB corrected) ($\times 10^6 M^{-1}s^{-1}$)	±Δ $k_{off,fast}$ (PB corrected) ($\times 10^6 M^{-1}s^{-1}$)
I4S8 -$preQ_1$	4.1	0.74	0.49	0.03	0.245	0.045	0.239	0.043
I4S8 +$preQ_1$	4.1	0.74	0.47	0.02	0.245	0.045	0.239	0.043

Supplementary Table S9 *Counts of short (represented in red) and long (represented in blue) binding events from the raster plot of 100 molecules for the two set of mutants with minimum SD-aptamer separation (I1S8) to maximum SD-aptamer separation (I6S8)*

	I1S8			***I6S8***		
Total Molecules	*# of short binding*	*# of long binding*	*Total binding*	*# of short binding*	*# of long binding*	*Total binding*
100	*190*	*112*	*302*	*651*	*119*	*770*

Supplementary Table S10 A *List of all unbound times ($t_{unbound}$) and association rates (k_{on}) for mutants with different SD-aptamer distance (I4S8 to I4S0)*

	$t_{unbound, slow}$ (s)	±Δ $t_{unbound,slow}$ (s)	A_1	±ΔA_1	$k_{on,slow}$ ($\times 10^6 M^{-1}s^{-1}$)	±Δ $k_{on,slow}$ ($\times 10^6 M^{-1}s^{-1}$)	$k_{on,slow}$ (PB corrected) ($\times 10^6 M^{-1}s^{-1}$)	±Δ $k_{on,slow}$ (PB corrected) ($\times 10^6 M^{-1}s^{-1}$)
**I4S8**	248.1	6.7	0.63	0.02	0.202	0.006	0.359	0.009
**I4S7**	284.3	6.3	0.60	0.02	0.176	0.004	0.334	0.007
**I4S6**	342.1	17.2	0.67	0.03	0.146	0.007	0.304	0.015
**I4S5**	369.4	30.4	0.63	0.01	0.135	0.011	0.297	0.024
**I4S0**	484.1	35.5	0.81	0.02	0.103	0.008	0.262	0.019

	$t_{unbound,fast}^{shared}$ (s)	±Δ $t_{unbound,fast}^{shared}$ (s)	A_2	±ΔA_2	$k_{on,fast}$ ($\times 10^6 M^{-1}s^{-1}$)	±Δ $k_{on,fast}$ ($\times 10^6 M^{-1}s^{-1}$)	$k_{on,fast}$ (PB corrected) ($\times 10^6 M^{-1}s^{-1}$)	±Δ $k_{on,fast}$ (PB corrected) ($\times 10^6 M^{-1}s^{-1}$)
**I4S8**	16.24	1.23	0.37	0.02	3.078	0.233	3.237	0.245
**I4S7**	16.24	1.23	0.40	0.02	3.078	0.233	3.237	0.245
**I4S6**	16.24	1.23	0.34	0.02	3.078	0.233	3.237	0.245
**I4S5**	16.24	1.23	0.37	0.01	3.078	0.233	3.237	0.245
**I4S0**	16.24	1.23	0.22	0.01	3.078	0.233	3.237	0.245

Supplementary Table S10 B *List of all bound times (t_{bound}) and dissociation rates (k_{off}) for mutants with different SD-aptamer distance (I4S8 to I4S0)*

	$t_{bound, slow}$ (s)	±Δ $t_{bound,slow}$ (s)	A_1	±ΔA_1	$k_{off,slow}$ ($\times 10^6 M^{-1}s^{-1}$)	±Δ $k_{off,slow}$ ($\times 10^6 M^{-1}s^{-1}$)	$k_{off,slow}$ (PB corrected) ($\times 10^6 M^{-1}s^{-1}$)	±Δ $k_{off,slow}$ (PB corrected) ($\times 10^6 M^{-1}s^{-1}$)
**I4S8**	114.9	43. 1	0.32	0.02	0.009	0.003	0.004	0.001
**I4S7**	53.6	4.1	0.30	0.02	0.019	0.001	0.014	0.001
**I4S6**	57.8	11.2	0.17	0.02	0.017	0.003	0.012	0.002
**I4S5**	131.7	42.6	0.45	0.03	0.008	0.002	0.003	0.001
**I4S0**	64.5	6.9	0.24	0.02	0.016	0.002	0.011	0.001

	$t_{bound,fast}^{shared}$ (s)	±Δ $t_{bound,fast}^{shared}$ (s)	A_2	±ΔA_2	$k_{off,fast}$ ($\times 10^6 M^{-1}s^{-1}$)	±Δ $k_{off,fast}$ ($\times 10^6 M^{-1}s^{-1}$)	$k_{off,fast}$ (PB corrected) ($\times 10^6 M^{-1}s^{-1}$)	±Δ $k_{off,fast}$ (PB corrected) ($\times 10^6 M^{-1}s^{-1}$)
**I4S8**	1.72	0.20	0.67	0.02	0.582	0.067	0.577	0.067
**I4S7**	1.72	0.20	0.70	0.02	0.582	0.067	0.577	0.067
**I4S6**	1.72	0.20	0.82	0.02	0.582	0.067	0.577	0.067
**I4S5**	1.72	0.20	0.54	0.03	0.582	0.067	0.577	0.067
**I4S0**	1.72	0.20	0.73	0.02	0.582	0.067	0.577	0.067

Supplementary Table S11 *Counts of short (represented in red) and long (represented in blue) binding events from the raster plot of 100 molecules for the two set of mutants with fully available SD-aptamer complementarity (I4S8) to no SD-aptamer complementarity (I4S0)*

	I4S8			***I4S0***		
Total Molecules	*# of short binding*	*# of long binding*	*Total binding*	*# of short binding*	*# of long binding*	*Total binding*
100	*527*	*81*	*608*	*253*	*45*	*248*

Supplementary Table S12 A *Unbound times ($t_{unbound}$) and associated rates (k_{on}) of 30S interaction with R-mRNA^{+30} under the influence of S1 protein*

	$t_{unbound,slow}$ (s)	±Δ $t_{unbound,slow}$ (s)	A_1	±ΔA_1	$k_{on,slow}$ (×$10^6 M^{-1}s^{-1}$)	±Δ $k_{on,slow}$ (×$10^6 M^{-1}s^{-1}$)	$k_{on,slow}$ (PB corrected) (×$10^6 M^{-1}s^{-1}$)	±Δ $k_{on,slow}$ (PB corrected) (×$10^6 M^{-1}s^{-1}$)
ΔS1-30S	622.3	1.85	1.27	0.00	0.080	0.001	0.239	0.001
ΔS1-30S+0.5x purified S1	605.9	2.09	1.06	0.00	0.083	0.001	0.241	0.001
ΔS1-30S+1x purified S1	531.3	1.80	0.93	0.00	0.094	0.001	0.252	0.001
WT-30S	299.9	35.26	0.90	0.04	0.167	0.019	0.325	0.038

	$t_{unbnd,fast}^{shared}$ (s)	±Δ $t_{unbound,fast}^{shared}$ (s)	A_2	±ΔA_2	$k_{on,fast}$ (×$10^6 M^{-1}s^{-1}$)	±Δ $k_{on,fast}$ (×$10^6 M^{-1}s^{-1}$)	$k_{on,fast}$ (PB corrected) (×$10^6 M^{-1}s^{-1}$)	±Δ $k_{on,fast}$ (PB corrected) (×$10^6 M^{-1}s^{-1}$)
ΔS1-30S	10.31	1.10	0.03	0.03	4.85	0.52	5.01	0.54
ΔS1-30S+0.5x purified S1	10.31	1.10	0.17	0.03	4.85	0.52	5.01	0.54
ΔS1-30S+1x purified S1	10.31	1.10	0.23	0.03	4.85	0.52	5.01	0.54
WT-30S	10.31	1.38	0.15	0.03	4.85	0.65	5.01	0.67

Supplementary Table S12 B *Bound times (t_{bound}) and dissociated rates (k_{off}) of 30S interaction with R-mRNA^{+30} under the influence of S1 protein*

	$t_{bound,slow}$ (s)	±Δ $t_{bound,slow}$ (s)	A_1	±ΔA_1	$k_{off,slow}$ (×$10^6 M^{-1}s^{-1}$)	±Δ $k_{off,slow}$ (×$10^6 M^{-1}s^{-1}$)	$k_{off,slow}$ (PB corrected) (×$10^6 M^{-1}s^{-1}$)	±Δ $k_{off,slow}$ (PB corrected) (×$10^6 M^{-1}s^{-1}$)
ΔS1-30S	66.8	0.6	0.25	0.001	0.015	0.001	0.010	0.001
ΔS1-30S+0.5x purified S1	68.3	0.3	0.53	0.001	0.015	0.001	0.009	0.001
ΔS1-30S+1x purified S1	103.2	0.3	0.64	0.001	0.009	0.001	0.005	0.001
WT-30S	137.2	41.3	0.58	0.084	0.007	0.002	0.002	0.003

	$t_{bound,fast}^{shared}$ (s)	±Δ $t_{bound,fast}^{shared}$ (s)	A_2	±ΔA_2	$k_{off,fast}$ (×$10^6 M^{-1}s^{-1}$)	±Δ $k_{off,fast}$ (×$10^6 M^{-1}s^{-1}$)	$k_{off,fast}$ (PB corrected) (×$10^6 M^{-1}s^{-1}$)	±Δ $k_{off,fast}$ (PB corrected) (×$10^6 M^{-1}s^{-1}$)
ΔS1-30S	7.22	1.10	0.74	0.001	0.138	0.021	0.133	0.020
ΔS1-30S+0.5x purified S1	7.22	1.10	0.40	0.003	0.138	0.021	0.133	0.020
ΔS1-30S+1x purified S1	7.22	1.10	0.27	0.003	0.138	0.021	0.133	0.020
WT-30S	7.22	1.10	0.36	0.034	0.138	0.021	0.133	0.020

Supplementary Table S13 A *Unbound times ($t_{unbound}$) and associated rates (k_{on}) for the influence of $preQ_1$ in absence and presence of initiation factors (IFs)*

	$t_{unbound,\ slow}$ (s)	±Δ $t_{unbound,slow}$ (s)	A_1	±ΔA_1	$k_{on,slow}$ (×10⁶M⁻¹s⁻¹)	±Δ $k_{on,slow}$ (×10⁶M⁻¹s⁻¹)	$k_{on,slow}$ (PB corrected) (×10⁶M⁻¹s⁻¹)	±Δ $k_{on,slow}$ (PB corrected) (×10⁶M⁻¹s⁻¹)
-IF; -$preQ_1$	299.9	35.3	0.89	0.04	0.167	0.019	0.325	0.038
-IF; +$preQ_1$	607.5	86.1	0.96	0.08	0.082	0.012	0.241	0.034
+IF; -$preQ_1$	220.3	7.2	0.76	0.03	0.227	0.007	0.385	0.013
+IF; +$preQ_1$	460.8	72.6	1.04	0.06	0.108	0.017	0.267	0.042

	$t_{unbound,fast}^{shared}$ (s)	±Δ $t_{unbound,fast}^{shared}$ (s)	A_2	±ΔA_2	$k_{on,fast}$ (×10⁶M⁻¹s⁻¹)	±Δ $k_{on,fast}$ (×10⁶M⁻¹s⁻¹)	$k_{on,fast}$ (PB corrected) (×10⁶M⁻¹s⁻¹)	±Δ $k_{on,fast}$ (PB corrected) (×10⁶M⁻¹s⁻¹)
-IF; -$preQ_1$	10.3	1.38	0.15	0.03	4.851	0.645	4.845	0.649
-IF; +$preQ_1$	10.3	1.38	0.22	0.01	4.851	0.645	4.845	0.649
+IF; -$preQ_1$	10.3	1.38	0.30	0.02	4.851	0.645	4.845	0.649
+IF; +$preQ_1$	10.3	1.38	0.07	0.01	4.851	0.645	4.845	0.649

Supplementary Table S13 B *Bound times (t_{bound}) and dissociated rates (k_{off}) for the influence of $preQ_1$ in absence and presence of initiation factors (IFs)*

	$t_{bound, slow}$ (s)	±Δ $t_{bound,slow}$ (s)	A_1	±ΔA_1	$k_{off,slow}$ (×$10^6 M^{-1}s^{-1}$)	±Δ $k_{off,slow}$ (×$10^6 M^{-1}s^{-1}$)	$k_{off,slow}$ (PB corrected) (×$10^6 M^{-1}s^{-1}$)	±Δ $k_{off,slow}$ (PB corrected) (×$10^6 M^{-1}s^{-1}$)
-IF; -$preQ_1$	137.2	41.3	0.58	0.08	0.007	0.002	0.002	0.001
-IF; +$preQ_1$	126.2	58.9	0.41	0.08	0.008	0.004	0.006	0.001
+IF; -$preQ_1$	98.8	13.9	0.42	0.04	0.010	0.001	0.005	0.001
+IF; +$preQ_1$	129.4	39.9	0.50	0.06	0.008	0.002	0.003	0.001

	$t_{bound,fast}^{shared}$ (s)	±Δ $t_{bound,fast}^{shared}$ (s)	A_2	±ΔA_2	$k_{off,fast}$ (×$10^6 M^{-1}s^{-1}$)	±Δ $k_{off,fast}$ (×$10^6 M^{-1}s^{-1}$)	$k_{off,fast}$ (PB corrected) (×$10^6 M^{-1}s^{-1}$)	±Δ $k_{off,fast}$ (PB corrected) (×$10^6 M^{-1}s^{-1}$)
-IF; -$preQ_1$	7.2	1.10	0.36	0.03	0.138	0.021	0.133	0.020
-IF; +$preQ_1$	7.2	1.10	0.58	0.08	0.138	0.021	0.133	0.020
+IF; -$preQ_1$	7.2	1.10	0.57	0.04	0.138	0.021	0.133	0.020
+IF; +$preQ_1$	7.2	1.10	0.46	0.06	0.138	0.021	0.133	0.020

REFERENCES

1. Verveer, P.J. and Bastiaens, P.I.H. (2003) Evaluation of global analysis algorithms for single frequency fluorescence lifetime imaging microscopy data. *J. Microsc.*, **209**, 1-7.
2. Verveer, P.J., Squire, A. and Bastiaens, P.I.H. (2000) Global Analysis of Fluorescence Lifetime Imaging Microscopy Data. *Biophys. J.*, **78**, 2127-2137.
3. Duss, O., Stepanyuk, G.A., Grot, A., O'Leary, S.E., Puglisi, J.D. and Williamson, J.R. (2018) Real-time assembly of ribonucleoprotein complexes on nascent RNA transcripts. *Nat. Commun.*, **9**, 5087.
4. Rinaldi, A.J., Lund, P.E., Blanco, M.R. and Walter, N.G. (2016) The Shine-Dalgarno sequence of riboswitch-regulated single mRNAs shows ligand-dependent accessibility bursts. *Nat. Commun.*, **7**, 8976.
